# Synchronization of pancreatic islets by periodic or non-periodic muscarinic agonist pulse trains

**DOI:** 10.1371/journal.pone.0211832

**Published:** 2019-02-06

**Authors:** Joel E. Adablah, Ryan Vinson, Michael G. Roper, Richard Bertram

**Affiliations:** 1 Department of Chemistry and Biochemistry, Florida State University, Tallahassee, Florida, United States of America; 2 Department of Mathematics, Florida State University, Tallahassee, Florida, United States of America; 3 Department of Mathematics and Programs in Molecular Biophysics and Neuroscience, Florida State University, Tallahassee, Florida, United States of America; International University of Health and Welfare School of Medicine, JAPAN

## Abstract

Pulsatile insulin secretion into the portal vein from the many pancreatic islets of Langerhans is critical for efficient glucose homeostasis. The islets are themselves endogenous oscillators, but since they are not physically coupled it is not obvious how their oscillations are synchronized across the pancreas. It has been proposed that synchronization of islets is achieved through periodic activity of intrapancreatic ganglia, and indeed there are data supporting this proposal. Postganglionic nerves are cholinergic, and their product, acetylcholine, can influence islet β-cells through actions on M_3_ muscarinic receptors which are coupled to G_q_ type G-proteins. In addition, the neurons secrete several peptide hormones that act on β-cell receptors. The data supporting synchronization via intrapancreatic ganglia are, however, limited. In particular, it has not been shown that trains of muscarinic pulses are effective at synchronizing islets in vitro. Also, if as has been suggested, there is a ganglionic pacemaker driving islets to a preferred frequency, no neural circuitry for this pacemaker has been identified. In this study, both points are addressed using a microfluidic system that allows for the pulsed application of the muscarinic agonist carbachol. We find that murine islets are entrained and synchronized over a wide range of frequencies when the carbachol pulsing is periodic, adding support to the hypothesis that ganglia can synchronize islets in vivo. We also find that islet synchronization is very effective even if the carbachol pulses are applied at random times. This suggests that a neural pacemaker is not needed; all that is required is that islets receive occasional coordinated input from postganglionic neurons. The endogenous rhythmic activity of the islets then sets the frequency of the islet population rhythm, while the input from ganglia acts only to keep the islet oscillators in phase.

## Introduction

The rodent endocrine pancreas contains thousands to tens of thousands of islets of Langerhans [1], while a human may have over 3 million [2]. Islets contain 500–2000 cells, with each cell type releasing particular hormones in a glucose-dependent manner [3]. The majority of these cells are insulin-secreting β-cells that, like other cells, metabolize glucose leading to an increase in the ratio of ATP/ADP. Unlike most other cells, β-cells express K_ATP_ ion channels that are inactivated by the increased ATP/ADP ratio, and the resulting decrease in hyperpolarizing K^+^ current depolarizes the cell membrane [4]. This depolarization opens voltage-gated Ca^2+^ channels allowing for the influx of Ca^2+^ that triggers insulin secretion into the blood [5,6]. Insulin promotes the absorption of glucose into fat, skeletal muscle, and liver cells [7].

Insulin release from individual islets is not static, but oscillates with a period of 3–7 min [8–11]. While the mechanism behind the oscillatory activity from single islets continues to be investigated [6,8,12], it has been shown that oscillations of insulin are also observed *in vivo* in many species [13–18]. Furthermore, it has been demonstrated that the action of insulin on target tissues is far more productive when its secretion pattern is pulsatile as opposed to static [19–21], and that individuals with Type 2 diabetes and their first degree relatives show disordered oscillations [22].

While the mechanisms underlying the oscillatory activity of single islets have been widely discussed, less work has been done on the mechanism or mechanisms through which islet oscillations are synchronized throughout the pancreas. One hypothesis is that islet oscillations are coordinated by neural stimuli from intrapancreatic ganglia [18,23–26]. This hypothesis is based on the rich innervation of the pancreas by preganglionic vagal neurons, some of which synapse onto intrapancreatic ganglia that are found in close proximity to islets [27–34]. These postganglionic nerves then provide innervation to islets [35–38].

Is this ganglionic innervation responsible for setting the period of insulin oscillations *in vivo*? This certainly seems possible. If this is the case, then the most likely neurotransmitter responsible for the islet synchronization would be acetylcholine (ACh). ACh is the primary parasympathetic neurotransmitter of postganglionic neurons in the pancreas and acts on β-cells via muscarinic G protein-coupled membrane receptors [24,39,40]. *In vitro* studies have shown that application of a short bolus of ACh, or the cholinergic agonist carbachol (CCh), is capable of transiently synchronizing glucose-induced Ca^2+^ oscillations in islets [25,26,41]. However, it has not been established whether a pulse train of ACh or CCh can maintain the synchronization of islets, nor if there are any constraints on the pulsing frequency to maintain any synchronization. We do this in the present study, using a previously described microfluidic device system [42,43] modified to allow for the application of a controlled pattern of CCh pulses.

Acetylcholine and CCh bind to M_3_ muscarinic receptors in murine islets, inducing the phospholipase C (PLC)-catalyzed hydrolysis of phospholipid phosphatidylinositol-4,5-bisphosphate (PIP_2_) producing inositol-1,4,5-trisphosphate (IP_3_) and diacylglycerol. Ca^2+^ is then mobilized from the IP_3_-sensitive stores of the endoplasmic reticulum (ER) thereby increasing the intracellular Ca^2+^ concentration ([Ca^2+^]_i_). This second messenger acts at several key points in the glucose transduction pathway that can influence islet oscillations, as well as stimulating insulin exocytosis [5,8]. Isolated murine β-cells and islets show a spike in [Ca^2+^]_i_ that promptly follows cholinergic stimulation in a glucose-rich environment [25,26,41,44,45]. This [Ca^2+^]_i_ spike can potentially reset islet oscillators, and thereby play a key role in the synchronization of islet oscillations. In this study, we use mathematical modeling to demonstrate how this resetting can be achieved, even in a model where factors in addition to Ca^2+^ are involved in the production of islet oscillations.

One study of the electrical properties of parasympathetic ganglia in a feline pancreas showed long recordings from ganglion cells that exhibited spontaneous electrical activity. Remarkably, this activity included bursts every 6 to 8 minutes, which is similar to the period of insulin oscillations *in vivo* (~5 min) [46]. This finding helped to establish the hypothesis that islets are synchronized through a periodic or almost-periodic neural signal provided by intrapancreatic ganglia. This proposal has been supported by other data, for example the intriguing and repeated observation that insulin levels remain pulsatile even in perfused pancreas preparations from a number of species [13–17], which suggests that the neural pacemaker resides in the ganglia themselves and not the brain. Also, rat islets transplanted into the liver produce a coherent pulsatile insulin pattern 200 days, but not 30 days, after transplantation [47], presumably after formation of an associated neural network innervating the islets [48].

In spite of these suggestive findings, it is not clear how such a neural pacemaker, located within distributed ganglia, would operate. Does each ganglion contain such a pacemaker? If so, how are they coordinated across ganglia? Is the pacemaker an emergent property of the network of ganglia? If so, how is the distributed circuitry organized to produce the pacemaking rhythm? These questions prompted us to ask a more basic question. Is periodic pacemaking even necessary to synchronize islets? Perhaps all that is required is that the islets receive coordinated signals from the ganglia to occasionally reset, and transiently synchronize, the islet oscillators. If this coordinated activity were sufficiently frequent, the islet oscillators could remain synchronized even though the synchronizing agent is not periodic. We tested this hypothesis with our microfluidic system by applying CCh pulses to islets with random timing. We found that this random pulse protocol was very effective at synchronizing islet Ca^2+^ oscillations. This *in vitro* data supports the idea that, *in vivo*, no neural pacemaker is necessary to maintain islet synchronization throughout the pancreas. Instead, islets only need to receive coherent input from the ganglia to occasionally reset them and thereby keep them synchronized.

## Materials & methods

### Chemicals & reagents

NaCl, CaCl_2_, MgCl_2_, tricine, DMSO, and antibiotic antimycotic solution (100X) were purchased from Sigma-Aldrich (Saint Louis, MO). NaOH and KCl were from EMD Chemicals (San Diego, CA). Glucose (dextrose), RPMI 1640, and gentamicin sulfate were from Thermo Fisher Scientific (Waltham, MA). Fura PE3 acetoxymethyl (AM) ester from Cayman Chemical (Ann Arbor, MI) was the Ca^2+^ reporter used. Pluronic F-127 was from Life Technologies (Grand Island, NY). Collagenase P (from *Clostrdium histolyticum*) was acquired from Roche Diagnostics (Indianapolis, IN). Cosmic Calf Serum was purchased from GE Healthcare Bio-Sciences (Pittsburgh, PA). Poly(dimethylsiloxane) (PDMS) base and curing agent were procured from Dow Corning (Midland, MI, USA). SU-8 2075 photoresist was from Microchem (Westborough, MA). All solutions were made with Milli-Q (Millipore, Bedford, MA, USA) 18 MΩ·cm ultrapure water. Glucose solutions were prepared with a buffered salt solution composed of 2.4 mM CaCl_2_, 125 mM NaCl, 1.2 mM MgCl_2_, 5.9 mM KCl, and 25 mM tricine.

### Microfluidic device and system

The PDMS-glass hybrid device was fabricated using conventional photolithography with SU-8 2075 photoresist. All fluidic channels were 250 x 40 μm (width x height). The device had 2 inputs connected to fluidic reservoirs: the first contained a solution of 11 mM glucose and the second contained 10 μM CCh dissolved in 11 mM glucose. These reservoirs were pressurized using a piezoelectric flow controller (Elvesys, Paris, France). Flow rates of the solutions were monitored and regulated by flow sensors (Elvesys, Paris, France) (see S1 Fig). The total flow rate of the solution delivered to islets was maintained at 1 μL/min ± 2% RSD. Islets were housed in a 0.6 mm diameter chamber that held 3–5 islets per experiment. In order to ensure physiological temperature within the islet chamber, 2 polyimide film heaters (Omega Engineering Inc., Norwalk, CT) connected to a CNi32 temperature controller (Omega Engineering Inc.) were attached underneath the device 1 mm away from the chamber. A thermocouple (Omega Engineering Inc.), sandwiched by the film heaters underneath the device, measured and maintained the chamber temperature throughout all experiments to be 36.5 °C (SD 0.5).

### Isolation, culture, and loading of islets of Langerhans

The isolation of islets from male CD-1 mice (30–40 g) was performed using collagenase P digestion as described previously [49]. All animal experiments were approved by the Florida State University Animal Care and Use Committee (protocol 1813). Islets from 2 mice per isolation batch were pooled together, incubated in RPMI 1640 supplemented with 11 mM glucose, L-glutamine, 10% Cosmic Calf Serum, 100 U/mL penicillin, 100 μg/mL streptomycin, and 10 μg/mL gentamicin at 37 °C and 5% CO_2_. Islets were used within 4 days after isolation. Prior to an experiment, a mixture of 1 μL of 5 mM Fura PE3-AM in DMSO and 1 μL of Pluronic F-127 in DMSO was added to 998 μL of RPMI 1640 to make 2.5 μM Fura PE3-AM. Randomly selected batches of 100–150 μm diameter islets were incubated in this solution for 40 min at 37 °C and 5% CO_2_. Islets were then manually loaded into the microfluidic chamber by sedimentation and rinsed with 11 mM glucose for 5 min prior to fluorescence measurement.

### Measurement of [Ca^2+^]_i_

The microfluidic device was secured onto the stage of a Ti-S inverted microscope (Nikon Instruments, Inc., Melville, NY). A Lambda XL lamp fitted with a shutter and filter wheel (Sutter Instruments, Novato, CA) containing appropriate emission filters was used to excite intracellular Fura PE3-AM at 340 and 380 nm. Fluorescence images were collected with a CCD camera (Cascade, Photometrics, Tucson, AZ) controlled by Nikon NIS Elements software (Nikon) every 20 s after a 150 ms exposure (S1 Fig). The ratio of fluorescence emission at 520 nm after the 340 nm excitation to that after 380 nm, F_340_/F_380_, was used to calculate [Ca^2+^]_i_ for each islet using calibration constants [50,51] that were calculated previously using a Ca^2+^ calibration kit [52].

### Pulse profiles & data analysis

Throughout all experiments, a total of 67 islets were used. In each experiment, 3–4 islets were placed in the microfluidic system and perfused with 11 mM glucose. After this initial perfusion, a variety of CCh profiles were applied, each consisting of a square-wave pulse train. The pulse trains were characterized by the *rest time* (R) between the pulses, the *concentration* (C) of the pulses, and the *duration* (D) of each pulse [53]. The value of C and D were held constant throughout these experiments at 10 μM and 10 s, respectively, while R was varied as described in the text. The experiments were repeated with additional groups of 3–4 islets. The figures show representative traces from one group of islets, while data from the other groups are shown in the Supplementary Figures, as noted in the text.

For data analysis, the average islet [Ca^2+^]_i_ trace from a single group was background subtracted using a linear fit to the data and smoothed by inserting two data points between each pair of points using linear interpolation. A spectrogram of the background-subtracted data was then produced using a custom LabVIEW program (National Instruments, Austin, TX) [52]. Briefly, a short-time Fourier transform (STFT) was performed on a 256-point Hanning window of the trace which produced an output with 1000 frequency bins. The window was then shifted by 5 data points and a new set of 256 points was analyzed. S2 Fig provides a summary of this workflow and an example of a spectrogram from a sine wave with variable frequency and amplitude. The spectrogram plots the period of the wave, determined by the STFT, as a function of experimental time with the magnitude of the peaks in the STFT shown in pseudocolor using the scale bar on the right. Use of this visual tool in the analysis of [Ca^2+^]_i_ data helps to quickly, yet quantitatively, determine the extent of synchronicity among islet groups as well as how the average islet [Ca^2+^]_i_ oscillation period changes in response to CCh. All spectrograms shown throughout the Results are on the same intensity scale.

To supplement this analysis, the major oscillation period of each islet is plotted before and after the initiation of pulsing. To identify the major oscillation period, fast Fourier transforms were performed on the individual islet [Ca^2+^]_i_ traces. The frequency with the highest magnitude was recorded before and after CCh pulses were applied and converted to an oscillation period (min). Calculated averages are reported with 1 SD in parentheses, unless otherwise noted.

### Mathematical modeling

**Fig 1:**
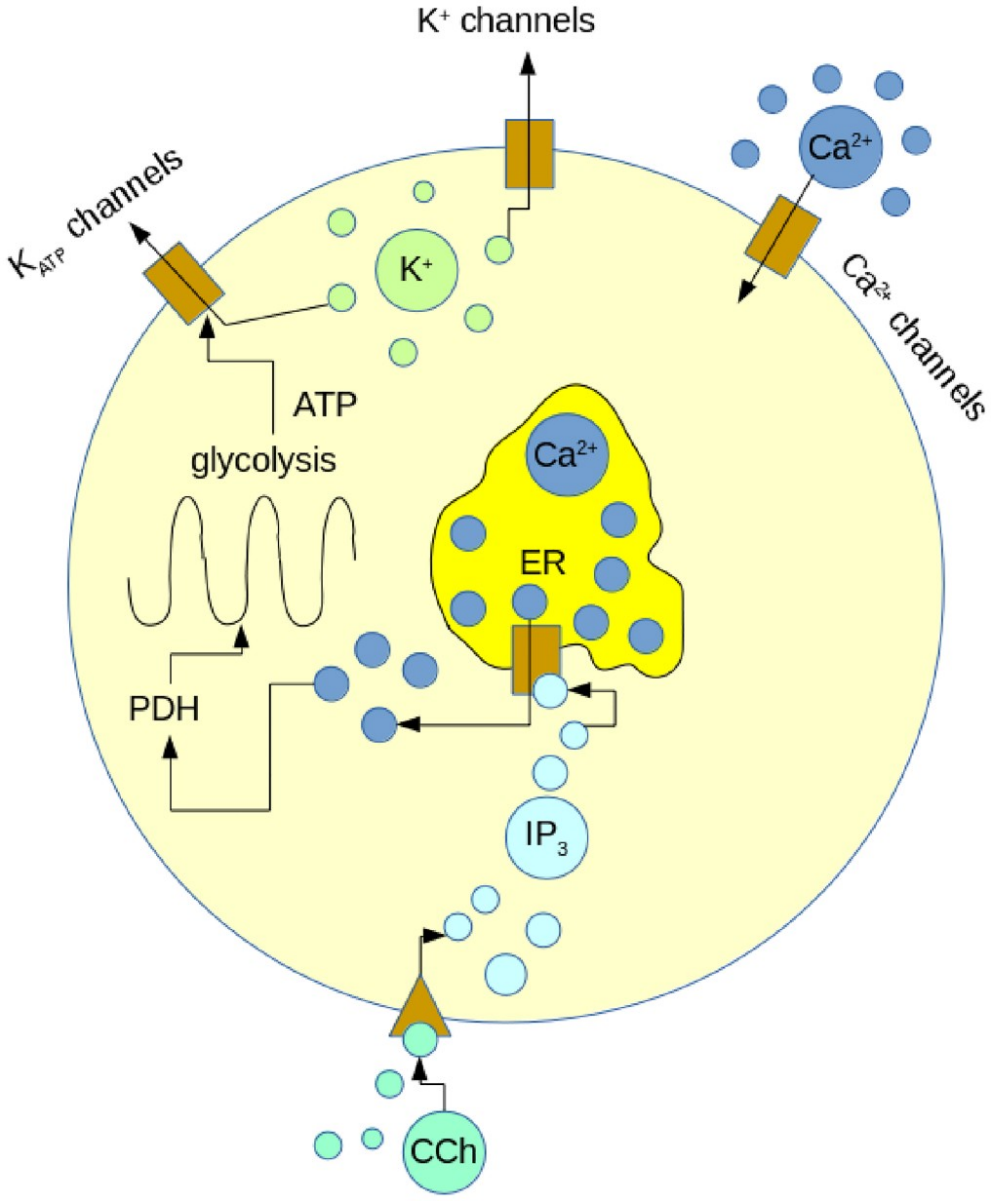
Diagram of the Integrated Oscillator Model (IOM). PDH: pyruvate dehydrogenase, IP_3_: inositol-1,4,5-trisphosphate, ER: endoplasmic reticulum. A representative view of the modified IOM model in which a key point is that intracellular Ca^2+^ acts on metabolism through activation of PDH. In this modified version of the IOM, external CCh pulses generate intracellular pulses of IP_3_.

The mathematical model is based on the Integrated Oscillator Model (IOM) introduced previously [6] and illustrated in Fig 1. The model consists of 7 ordinary differential equations for the membrane potential, a delayed rectifying K^+^ channel activation variable, concentration of free cytosolic Ca^2+^, free Ca^2+^ in the ER, fructose 6-phosphate, fructose 1,6-bisphosphate (FBP), and ATP. It describes 2 coupled conditional oscillators: a glycolytic oscillator and an electrical oscillator. Coupling is bidirectional: ATP produced through metabolism inhibits K_ATP_ channels in the plasma membrane, depolarizing the membrane and thereby influencing the electrical oscillator, while Ca^2+^ that enters through plasma membrane Ca^2+^ channels, or that is released from the ER, affects glycolysis via activation of pyruvate dehydrogenase (PDH). The IOM can simulate oscillations in electrical activity and Ca^2+^ dynamics with a period of approximately 5 min, as observed experimentally. It is assumed that a model cell is representative of all cells in an islet, which are coupled together and synchronized through gap junctions. See elsewhere [6,42] for more details.

The IOM was modified to simulate the effects of activation of muscarinic acetylcholine receptors by CCh. Activated receptors result in the production of IP_3_ from PIP_2_ via the enzyme phospholipase C. Upon binding to IP_3_ receptors, Ca^2+^ is released from the ER (Fig 1). We include this step of the pathway in our model, treating a pulse of CCh as a pulse of IP_3_ production with amplitude of IP_3amp_. The intracellular concentration of IP_3_ ([IP_3_]) is then described by the following differential equation:

$$\frac{d[{IP}_{3}]}{dt}\;=\;\frac{{IP}_{3amp}-[{IP}_{3}]}{\tau_{{IP}_{3}}}$$ (1)

where *IP*_3 *amp*_ = 0.35 μM when the 10 sec pulse was on, and 0 otherwise. The parameter $\tau_{{IP}_{3}}\;=\;10\;$ sec is a time constant that sets the speed at which the IP_3_ concentration changes. The code for the model is available for free from www.math.fsu.edu/~bertram/software/islet.

## Results

The microfluidic platform employed for this study facilitated precise and automated delivery of glucose and the various CCh profiles to groups of 3–4 islets. Multiple experiments were performed to investigate the effects of different CCh profiles. Although the layout of the microfluidic channels has been described previously, the use of the piezoelectric transducer in conjunction with the flow rate sensors was not. We found that this active flow rate control was much better at rapidly generating pulses and maintaining stable flow rates than passive methods. The reagents took 48 s (SD 1) to travel the 75 mm long channel from the inputs to the islet chamber. This dispersive flow resulted in attenuation of the 10 μM CCh pulses by 43.5% (SD 0.4). Thus, the concentration felt by the islets was ~5.7 μM. This value is lower than the those used in other reports [25,26] although, as shown below, it was still large enough to induce responses in all islets tested.

### Islets are unsynchronized with application of constant 11 mM glucose

Control experiments were performed by continuously perfusing 3 groups of islets (S3–S5 Figs) with 11 mM glucose to test if they would synchronize in the absence of CCh. There was no obvious synchronization among islets over 90 minutes of continuous Ca^2+^ measurement from the 3 different experimental groups (n = 11 total islets) tested. Any observed synchronization in the average Ca^2+^ trace was attributed to the small islet number within a single group.

### Islets are entrained by periodic CCh pulses with a rest time of R = 2 min

**Fig 2:**
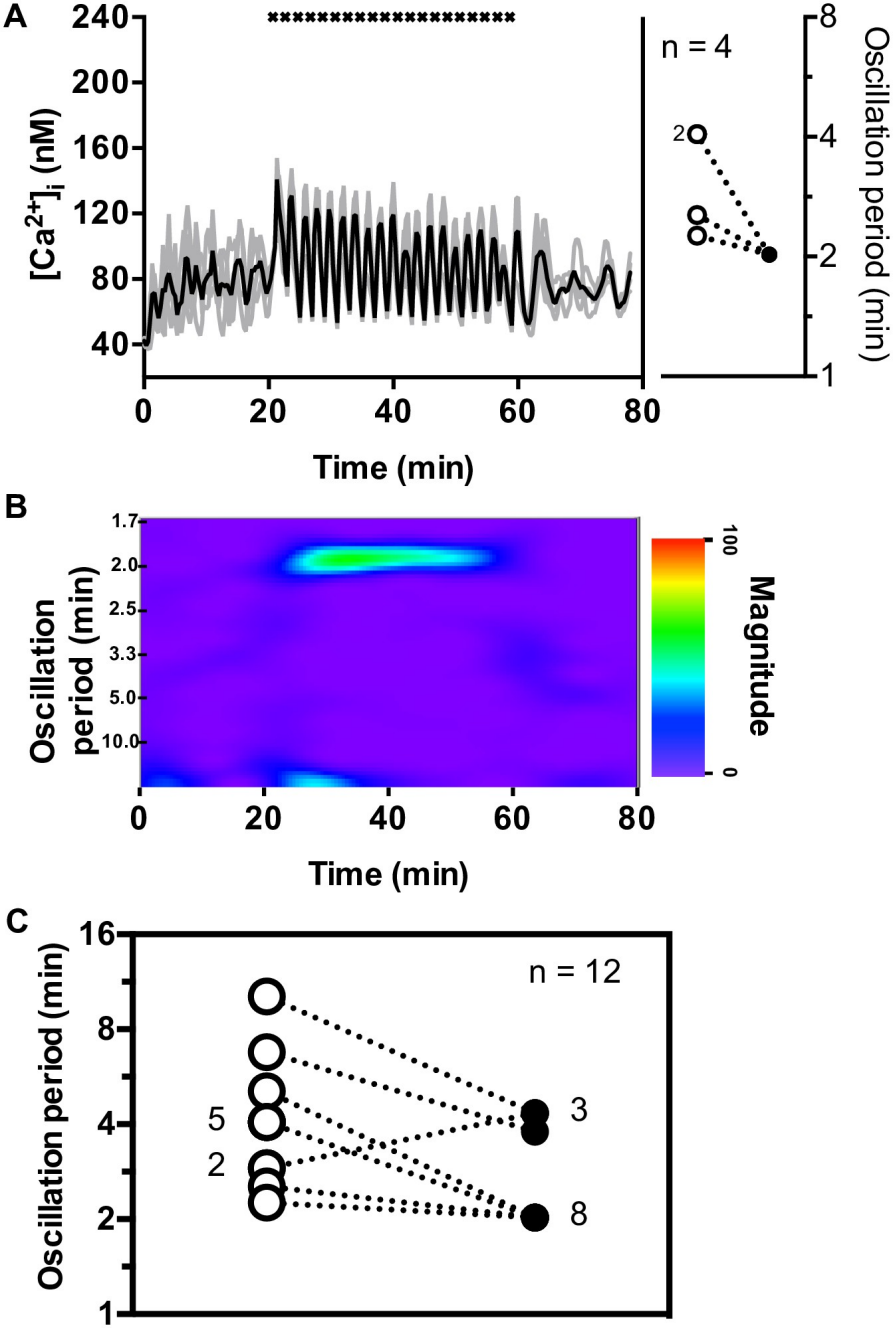
Effect of CCh pulses with rest time of R = 2 min on [Ca^2+^]_i_ oscillations. (A) The [Ca^2+^]_i_ traces (gray lines) of four islets and their average (black line) are shown when CCh pulses with rest durations of R = 2 min were applied. The timing of the CCh pulses is shown by the “x” at the top of the figure. The inset shows all four islets had a natural oscillation period between 2 and 4 min (open circles) prior to CCh pulsing, which changed to 2 min upon pulsing (filled circles) indicating 1:1 entrainment. (B) The spectrogram of the average [Ca^2+^]_i_ from the 4 islets in (A) is shown. As can be seen, while there was no major oscillation period prior to pulsing, a strong band appeared during the 38 min that CCh was applied showing synchronicity by the group at an oscillation period of 2 min. The band at the bottom of the spectrogram at the beginning of the pulsing is an artifact from the data analysis. See S6 and S7 Figs for results from the remaining R = 2 min groups. (C) The oscillation periods of all 12 islets tested are shown prior to (open circles), and during R = 2 min CCh pulsing (filled circles).

We next challenged three groups of islets (four islets in each group) with a train of twenty CCh pulses with R = 2 min, i.e., 2 min between each pulse. Fig 2A shows Ca^2+^ oscillations of individual islets (gray traces) from one of the three groups as well as the mean of the four islets (black trace). Before CCh pulsing commenced, the Ca^2+^ oscillations were out of phase as shown by the minimal rhythmicity in the mean trace. The inset of Fig 2A shows two islets had natural oscillation periods (open circles) of 4.1 min. The other two had periods of 2.3 and 2.5 min. Once CCh pulses began, the endogenous Ca^2+^ oscillations were dominated by the effects of the cholinergic pulses; all four of the islets shifted their oscillation periods to 2.0 min indicating 1:1 entrainment by the periodic CCh pulses (filled circles, Fig 2A inset). The mean [Ca^2+^]_i_ trace shows clear oscillations with a frequency that matches that of the CCh pulses. The spectrogram (Fig 2B) of the average Ca^2+^ trace in Fig 2A shows clear synchronization among the islets with the emergence of a strong band at an oscillation period of 2 min that lasted the duration of the CCh pulsing.

S6 Fig shows a similar 1:1 entrainment pattern of a second group of four islets with natural oscillation periods of either ~4 or ~5 min. The remaining four islets (S7 Fig) exhibited a pattern closer to 2:1 entrainment, with the frequency of the CCh pulsing twice that of the Ca^2+^ oscillations. Two of these islets had long natural oscillation periods of 6.8 and 10.2 min and the stimulus train made them oscillate faster, though the entrained period (~4 min) was still about twice that of the stimulus train. In contrast, for the remaining two islets, the entrained period was longer than that of their natural periods (~2.9 min). Fig 2C summarizes the oscillation period transitions for all 12 islets challenged with R = 2 min pulses. Thus, the stimulus train can either slow down or speed up islet oscillations so that the population becomes synchronized.

### Islets are entrained by periodic CCh pulses with a rest time of R = 5 min

**Fig 3:**
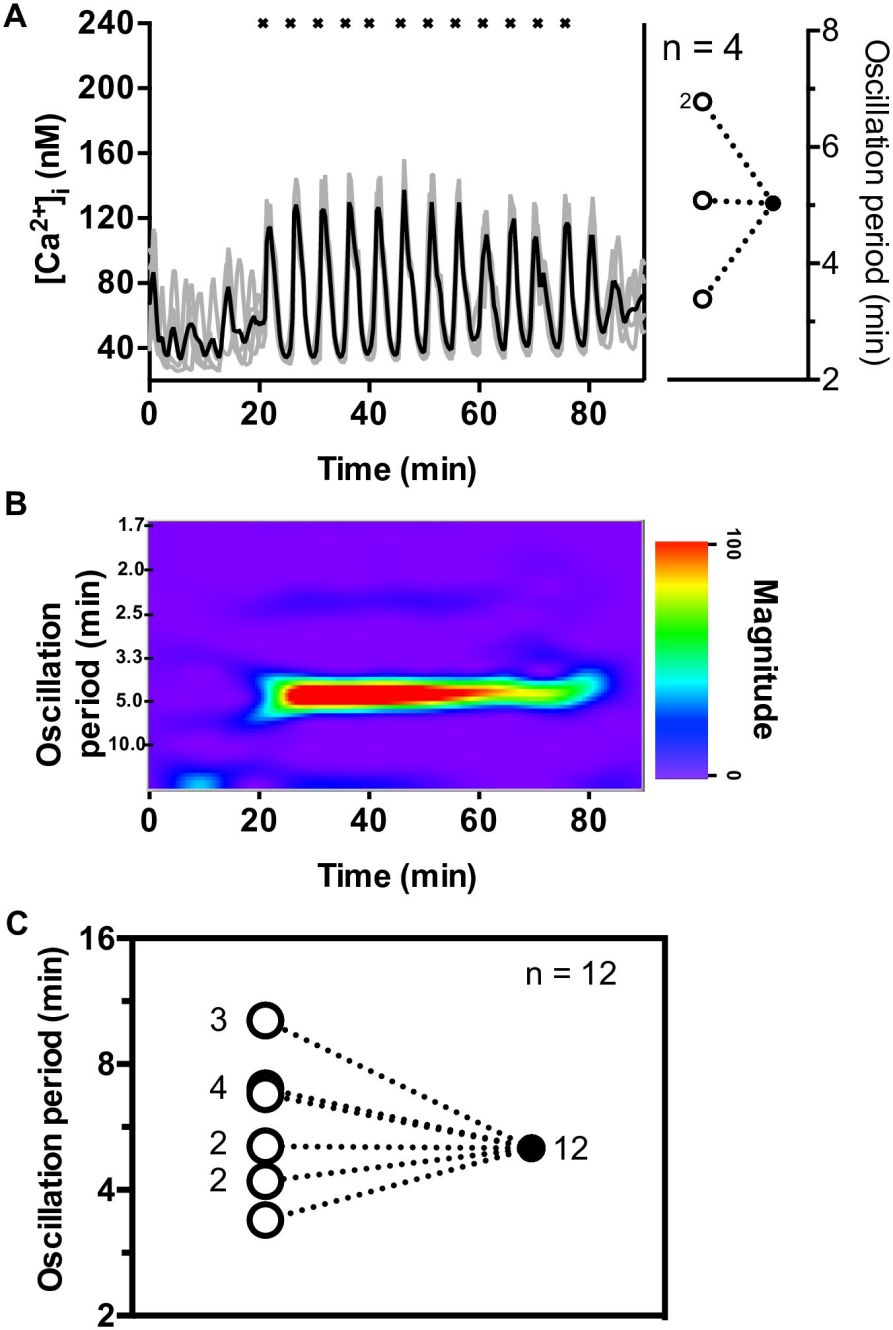
Effect of CCh pulses with rest time of R = 5 min on [Ca^2+^]_i_ oscillations. (A) The individual [Ca^2+^]_i_ from all 4 islets are shown in gray and their average is shown in black. The timing of the CCh pulses is shown by the “x”. The inset shows the natural period of these 4 islets ranged from 3.4–6.8 min (open circle), but all converged to 5 min during pulsing (filled circle) indicating 1:1 entrainment. (B) The synchronized response of the group is evident by the emergence of a pronounced band at an oscillation period of 5 min during the 55 min time that CCh was applied. See S8 and S9 Figs for results from the remaining R = 5 min groups. (C) The oscillation periods of all 12 islets tested with the R = 5 min pulse profile before (open circles) and during CCh pulsing (filled circles) are summarized.

Another set of experiments was performed with three groups of islets (four islets per group) using R = 5 min. As with previous experiments, islets were not synchronized prior to the application of cholinergic pulses. However, all groups were immediately entrained once the periodic CCh pulses commenced. The four islet average [Ca^2+^]_i_ trace in the representative experiment is shown in Fig 3A with distinct oscillations that matched the frequency of the CCh stimulation. As the inset illustrates, the natural oscillation periods for these 4 islets were 3.4, 5.1, or 6.8 min and they all exhibited 1:1 entrainment during pulsing. This highly coordinated behavior is shown in the spectrogram (Fig 3B) with the emergence of a prominent band at an oscillation period of 5.0 min which fades away soon after the final CCh pulse is applied. S8 and S9 Figs show the other two groups of islets exhibiting 1:1 entrainment by the 5 min train of CCh pulses. Fig 3C summarizes the transitions of the oscillation periods from all 3 islet groups tested with this pulse profile. Although one of the groups (S8 Fig) did not have a robust signal in the spectrogram, this was due to the weaker [Ca^2+^]_i_ peaks measured from this group. Nevertheless, it is clear from the analysis of the average [Ca^2+^]_i_ trace and the change in oscillation periods of the individual islets that the islets were entrained 1:1. It is notable that the 5 min periodicity of the CCh pulse train, so effective at entraining the islets, is similar to that of blood insulin oscillations in healthy animals [13–18].

### Islet entrainment is variable with R = 10 min carbachol pulses

**Fig 4:**
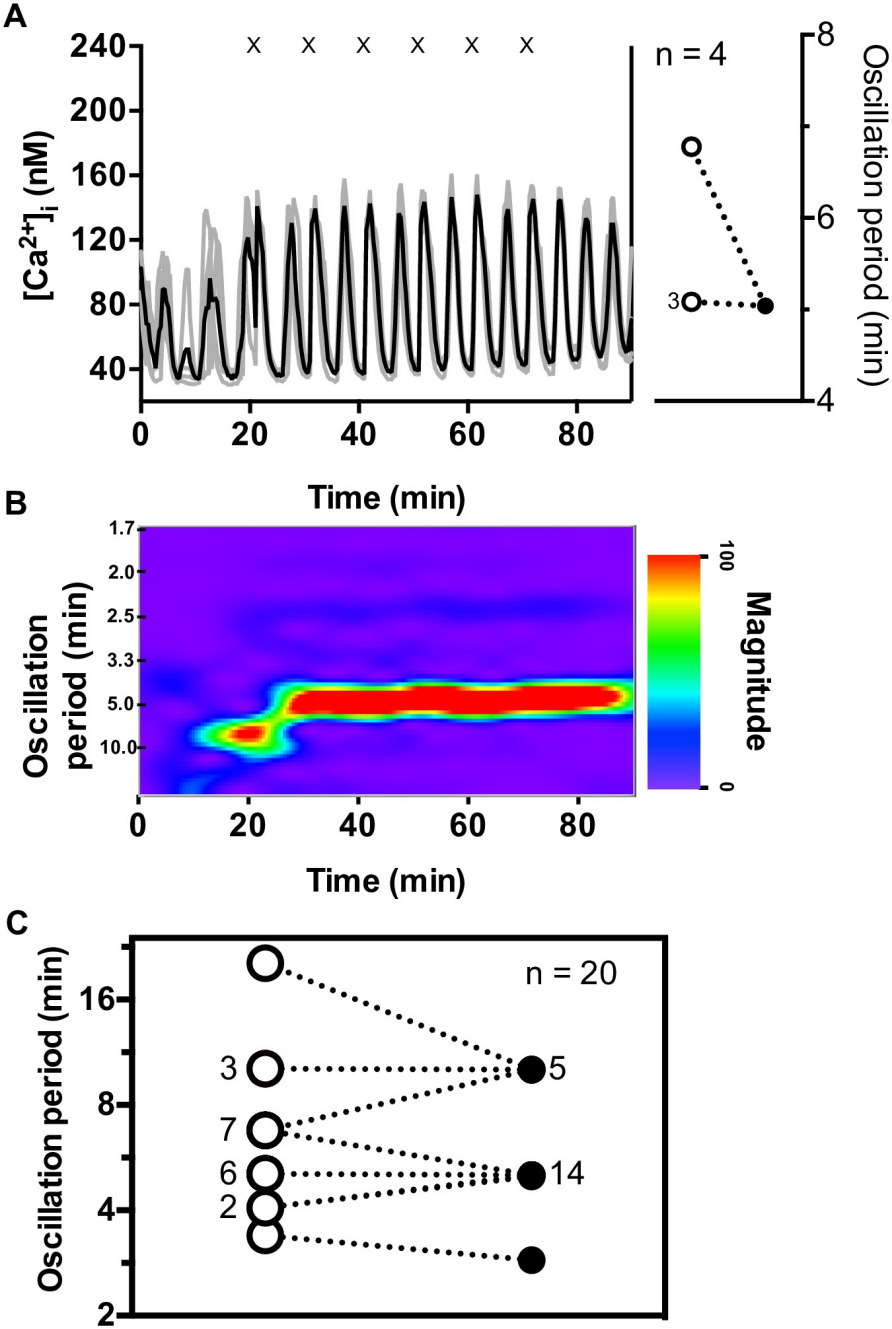
Effect of CCh pulses with rest time of R = 10 min on [Ca^2+^]_i_ oscillations. (A) In this example, all four islets had a natural period, as seen in the inset, between 5.1–6.8 min (open circles) which entrained to 5 min (1:2) during the pulsing period (filled circle). The number of islets with identical oscillation periods before or during pulsing is indicated adjacent to the relevant circle. (B) The synchronized response of the group is evident by the emergence of a pronounced band at 5 min during the CCh pulsing. After the final pulse, 5 min oscillations persisted until the end of the Ca^2+^ measurement. See S10–S13 Figs for results from the remaining R = 10 min groups. (C) A compilation of the oscillation periods of all 20 islets tested prior to (open circles) and during pulsing (filled circle) with R = 10 min is shown.

The next set of experiments used CCh pulses with R = 10 min, which is longer than the natural oscillation period found in 58 of the 67 islets investigated in this study. Five groups of islets were tested with CCh pulses using R = 10 min (four islets in each group). All groups showed entrainment, but with considerable variability in the profiles. The most common entrainment mode (n = 14 / 20 islets) was 1:2 (one CCh pulse for every two Ca^2+^ peaks) with all but one islet in this subpopulation having natural oscillation periods in the 4–7 min range. A representative group of this subpopulation is shown in Fig 4A with all four islets having natural oscillation periods of either 5.1 or 6.8 min transitioning into pronounced 5 min oscillations during pulsing. The high degree of synchronization is immediately apparent in the spectrogram (Fig 4B) by the intense band at a 5 min oscillation period.

The data for the four remaining groups of islets are shown in S10–S13 Figs with the majority of these islets showing 1:2 entrainment. The second most common entrainment mode was 1:1 (5 / 20 islets), and the majority of these islets had long natural periods prior to pulsing. An interesting case is shown in S13 Fig where islets showed natural oscillation periods of either 6.8 or 10.2 min. During CCh pulsing, the islets produced doublet Ca^2+^ oscillations that had a ~10 min gap between each corresponding peak in the doublet and a ~5 min gap between the doublet peaks. The 5 and 10 min bands clearly seen in the associated spectrogram show the synchronicity in this motif among these islets. Finally, one islet out of the population of twenty showed 1:3 entrainment by the 10 min CCh pulses. This islet (S12 Fig) had the shortest natural oscillation period of 3.4 min of all the islets tested in this set of experiments. Fig 4C shows the oscillation period transitions for all 20 islets before and during the challenge with R = 10 min pulsing.

### Model-generated phase response curves provide insight into entrainment

Phase Response Curves (PRCs) are often used to characterize the effect that an external pulse has on an oscillator. These curves show whether the phase of the oscillator is delayed or advanced in response to the pulse. This phase change typically depends on the initial phase of the oscillator when the pulse was applied. The PRC plot shows the phase change for each phase at which the pulse was applied. We generated a PRC for the CCh-induced changes in the islet oscillator using a mathematical model for β-cell oscillations, the IOM (see Methods). This allowed us to apply pulses at any phase during the oscillation and determine the degree to which it advanced or delayed the oscillator. It also allowed for comparison of perturbed with unperturbed Ca^2+^ oscillations. Rather than simulating the full CCh-induced signal transduction process, we modeled a pulse of IP_3_ generation at different phases of the intracellular Ca^2+^ oscillation, since IP_3_ is the product of CCh-receptor activation that induces release of Ca^2+^ from the ER.

**Fig 5:**
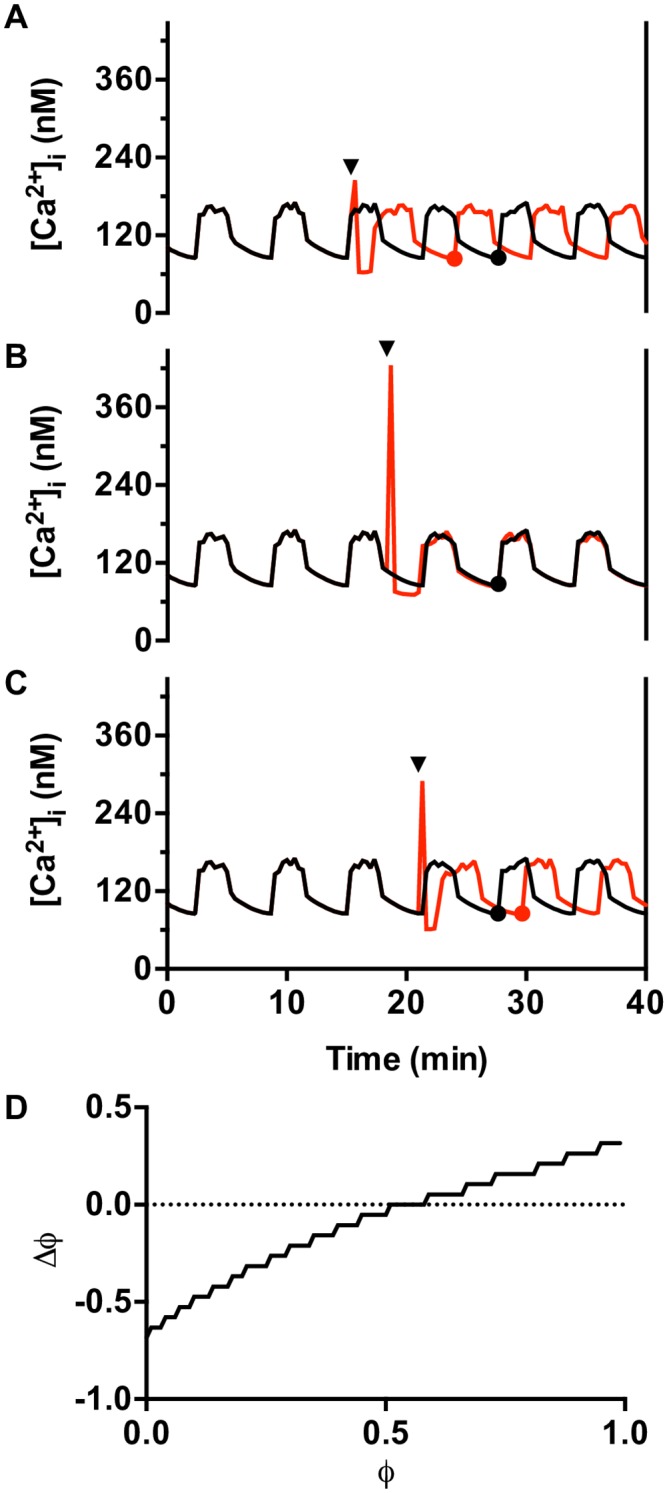
Phase responses of a model islet oscillator to a single pulse of IP_3_. Unperturbed model islet oscillations are shown in black. The red trace shows the model islet perturbed with a single 10 sec IP_3_ pulse applied at 20 min. (A) When applied during a burst active phase the IP_3_ pulse terminates the burst and there is an advance in the timing of the next active pase. (B) When applied early in a silent phase there is little effect on the phase of the islet oscillator. (C) When applied late in a silent phase there is a delay in the next burst. (D) A phase response curve showing the effect of an IP_3_ pulse on the phase of the islet oscillator. Phase 0 is the beginning of a burst active phase, while phase 1 is the end of the subsequent silent phase. The abscissa shows the phase at which the stimulus was applied, while the ordinate shows the effect the perturbation has on the phase as determined by the difference in timing of the unperturbed nadir (black circle in panels above) and the perturbed nadir (red circle). A negative phase difference means a phase advance, while a positive difference means a phase delay.

The construction of the PRC is shown in Fig 5. In Fig 5A, the Ca^2+^ oscillations that occurred in a model islet in the absence of an IP_3_ pulse are shown by the black trace. The red curve is for the same model islet, but with a pulse of IP_3_ initiated at the arrow. This pulse resulted in a transient increase in Ca^2+^, reflecting its release from the ER, followed by a rapid reduction. The generation of a new Ca^2+^ plateau indicates that the IP_3_ pulse reset the oscillation. As the nadir of this new oscillation (red circle) occurred before the nadir of the corresponding unperturbed oscillation (black circle), the IP_3_ pulse caused a phase advance in the islet oscillator. In Fig 5B, the IP_3_ pulse was generated later during the declining phase of the Ca^2+^ oscillations. There was again a transient increase in Ca^2+^ followed by a decline, after which followed a new Ca^2+^ plateau. The nadir of the reset Ca^2+^ oscillation coincided with the nadir of the unperturbed oscillation, indicating an unchanged phase by the IP_3_ pulse. Finally, in Fig 5C, the IP_3_ pulse was generated at the end of the silent phase, and the reset oscillation now had a nadir that occurred later than in the unperturbed model cell, indicating the phase was delayed.

The PRC in Fig 5D summarizes the effects of an IP_3_ pulse on the phase of the model islet oscillator. Here, the abscissa corresponds to a normalized Ca^2+^ oscillation with 0 reflecting the beginning of the Ca^2+^ plateau and 1 representing the end of the silent phase (i.e., the Ca^2+^ nadir). A negative and positive phase difference, Δϕ, represents a phase advance, and delay, respectively. When an IP_3_ pulse was given during a Ca^2+^ plateau, ϕ < 0.5, there was a phase advance. If given during the early part of the silent phase, approximately 0.5 < ϕ < 0.6, there was no phase shift. If given later in the silent phase, ϕ > 0.6, there was a phase delay. A PRC that exhibits both phase advances and phase delays is known as a type 2 PRC, and these have been shown to facilitate entrainment by periodic stimuli [54,55]. This result is consistent with the experimental results that showed islet entrainment by the CCh pulses.

### Periodic IP_3_ pulses entrain the model metabolic oscillations

**Fig 6:**
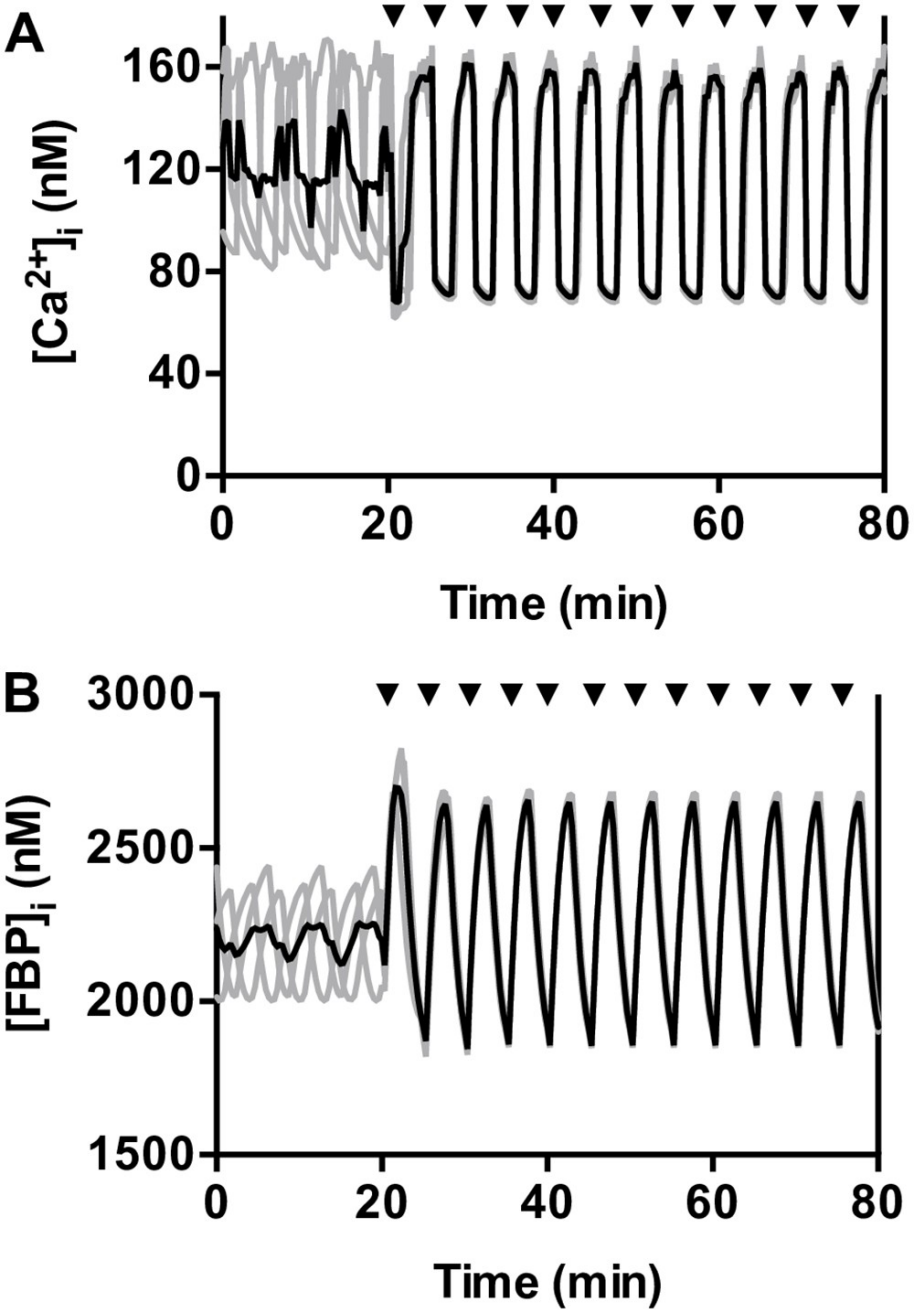
Model islets are synchronized by exposure to IP_3_ pulses with rest period R = 5 min. The time courses of the three model islets are in gray, and the mean is in black. An IP_3_ pulse is applied at each arrow. (A) Ca^2+^ traces quickly synchronize when IP_3_ stimulation begins. (B) The glycolytic metabolite FBP also synchronizes.

As expected from the type 2 PRC, the model islet can be entrained by IP_3_ pulses applied periodically, at least within an entrainment window. This is shown in Fig 6A, where three model islets are shown initially free-running. The model islets differ in their conductance values for Ca^2+^ (g_CaV_) and K_ATP_ (g_K(ATP)_) channels, and this heterogeneity results in differences in their natural oscillation period. Using a periodic application of 10 s IP_3_ pulses with R = 5 min, the model islets were quickly entrained and synchronized, similar to experimental results (Fig 3).

In the model, islet oscillations are due to bidirectional interactions between metabolism and intracellular Ca^2+^ (Fig 1 and [6]). Although IP_3_ pulses act only on the [Ca^2+^]_i_, and not directly on metabolism, this action is sufficient to entrain the metabolic oscillations and synchronize them across the three model islets. This is illustrated in Fig 6B, which shows the concentration of FBP for the three model islets in Fig 6A. FBP is the product of phosphofructokinase, a key allosteric enzyme in the glycolytic pathway. Oscillations in FBP reflect oscillations in downstream glycolytic and TCA cycle metabolites, and the end product, ATP [56]. The FBP oscillations were out of phase with one another before IP_3_ pulsing began, but quickly became entrained by the IP_3_ pulses. This entrainment is through the feedback of Ca^2+^ onto pyruvate dehydrogenase (Fig 1), which results in increased flux through glycolysis. Thus, the Ca^2+^ released from the ER with each IP_3_ pulse leads to an increase in metabolism, which influences the electrical activity through the actions of ADP and ATP on K_ATP_ channels. Since all model cells see the same periodic stimuli, and the PRC is type 2, these stimuli synchronize islet metabolism, electrical activity, and intracellular Ca^2+^ levels.

### Randomly-spaced CCh pulses synchronize [Ca^2+^]_i_ oscillations in islets and model islets

**Fig 7:**
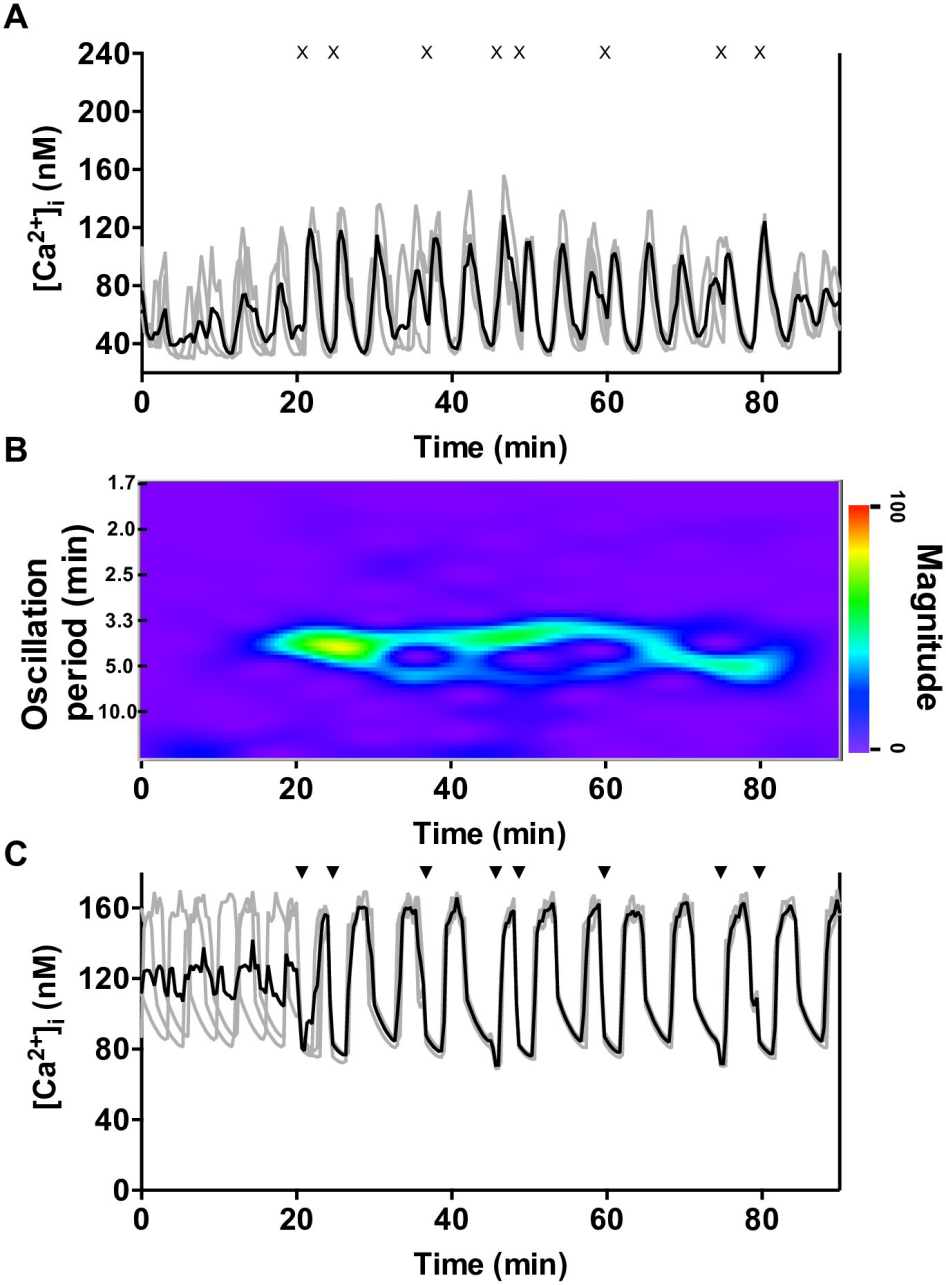
Synchronization of islets and model islets by randomly-spaced CCh pulses. **A.** The Ca^2+^ traces (gray lines) from a representative experiment with a set of 4 islets are overlaid with the average [Ca^2+^]_i_ (black line). The timing of the CCh pulses are shown by an “x” above the figure. B. Although randomly spaced, the repeated CCh pulses produced a synchronized population as can be seen from the emergence of a coherent band in the spectrogram. Synchronization decreased shortly after the final pulse at 79 min. See S14 and S15 Figs for the remaining two groups of islets stimulated with randomly timed pulses of CCh. C. Four model islets (grey lines) are stimulated by an identical pattern of IP_3_ pulses “▼” as the experiment shown in A. The resulting average (black line) demonstrates a synchronized population for the entire pulsing duration.

We have demonstrated that periodically-applied CCh pulses can entrain islets. We next set out to test if islets would still synchronize if there was no periodicity in the pulsing. Three experiments, each with four islets, were performed, with each group receiving a unique CCh pulse profile. In the representative example from one group (Fig 7), the average Ca^2+^ trace (A) and spectrogram (B) both highlight the poor synchronization of the islets during the 20 min prior to pulsing. After this time, a train of eight randomly spaced CCh pulses was applied where the R values between successive pulses were randomly selected integers from 2–18 min. When the pulses started, the islets quickly synchronized with the first pulse and remained mostly synchronized throughout the pulse train. The oscillation period of the average Ca^2+^ trace varied over time (as is evident in the spectrogram of Fig 7B), so it would be incorrect to say that the population average exhibited periodicity. This is to be expected, however, since the islets were not entrained by a periodic stimulus. However, there were strong oscillations in the average Ca^2+^ trace, indicating islet synchronization. After the final pulse was applied, the islets drifted out of phase and synchronization diminished. Results from the other two groups, shown in S14 and S15 Figs, again exhibit synchronization driven by randomly-timed CCh pulses.

One of the randomly-timed pulse profiles was simulated with four model islets and they showed similar behavior (Fig 7C). Although the [Ca^2+^]_i_ oscillations were initially out of phase, they quickly synchronized with the initiation of IP_3_ pulsing. The oscillations remained synchronized throughout the duration of the pulses, and only drifted apart once the pulsing was discontinued. Thus, as depicted by both experiments and model simulations, synchronization of [Ca^2+^]_i_ oscillations is possible without periodic exogenous CCh pulses.

## Discussion

We have demonstrated that periodic pulses of CCh are capable of entraining pancreatic islet Ca^2+^ oscillations with stimulus periods ranging from 2 to 10 min. There was a clear preference for a ~5 min response period, which is the natural period of most individual islet oscillators. Further, all islets subjected to the CCh pulses with a 5 min rest time responded with Ca^2+^ oscillations of 5 min periodicity. One third of the islets stimulated at a higher frequency (2 min rest time) responded by oscillating with a period of 4 min. Fourteen of the 20 islets stimulated at a low frequency (10 min stimulus period) responded with oscillations of 5 min period, due to 1:2 entrainment. It is evident, then, that a cholinergic agonist like CCh, or the related ACh, could be an effective islet synchronizer if applied periodically.

Prior studies have reported on the effects of islet [Ca^2+^]_i_ when using a single pulse of ACh [41,44,45] or CCh [25,26]. In the latter studies, it was shown that a single CCh pulse can synchronize islets for at least 40 min after the pulse is applied. Although we did not observe such a long-lasting effect from a single CCh pulse, we did find repeated CCh pulses to have a very strong entraining effect. One caveat to all of these in vitro reports is that it is not known how representative the in vitro pulse profiles are to the physiological state. The pulses delivered in our studies (10 s pulses with ~5 μM concentration experienced by islets) were shorter and of lower concentration than what others have used for the single pulse experiments.

Could periodic cholinergic stimulation occur *in vivo*, and if so, what would be the mechanism? One possibility is that there is coordinated periodic release of ACh from intrapancreatic ganglia. These ganglia were first reported by Langerhans in 1869 [57] and consist of clusters of neurons that spread in a connective plexus throughout the pancreas of several animal species [24,27–33] including humans [34]. This intrapancreatic ganglia network is innervated by preganglionic vagal neurons, although this input is not required to maintain activity of the ganglia since evoked and spontaneous electrical activity has been recorded in perfused pancreas [46] and intrapancreatic ganglia remain viable long after external pancreatic denervation [57]. For this viability to be maintained the ganglia must function without external stimulation. In rodents, ACh appears to be primarily a neural signal released by postganglionic neurons in close proximity to islets [45]. In humans, one pair of studies found that there is minor autonomic neural innervation of islets [58,59]. However, human islet α-cells appeared to be cholinergic, suggesting that acetylcholine acts mainly as a paracrine factor on both β-cells and δ-cells [45,58]. A more recent study using different methodology, 3D histology with tissue clearance, found the opposite; the study showed extensive parasympathetic innervation into the islet core, and intrapancreatic ganglia filled with vesicular ACh transporter varicosities, while α-cells were negative for ACh transporter staining [34].

An intriguing and repeated observation is that insulin levels remain pulsatile even in perfused pancreas preparations from a number of species [13–17]. Also, rat islets transplanted into the liver produce a coherent pulsatile insulin pattern 200 days, but not 30 days, after transplantation [47], presumably after formation of an associated neural network innervating the islets [48]. Indeed, these observations have driven much of the speculation that islets are synchronized through an intrapancreatic nervous system. The thought is that the intrapancreatic neural network acts as a pacemaker, much as the sinoatrial node does in the heart, periodically delivering pulses of ACh, peptide neurotransmitters, or ATP to islets and thereby entraining them (see [24] for a review). This hypothesis is supported by long recordings of ganglia nerve electrical activity, showing spontaneous bursts of activity with periodicity of 6–8 min [46]. We have demonstrated, though, that islet synchronization can be achieved even if the entraining signal is not periodic (Fig 7). This suggests that the intrapancreatic neural network need not be a pacemaker at all, but just needs to send coherent stimulation to the islets at random times as long as the stimuli are not too far apart in time. In this way, the stimuli would not be entraining the islets, but just occasionally coordinating them. The natural 5 min periodicity of most of the islet oscillators would then be sufficient to provide the rhythm underlying the observed insulin pulsatility. The neural circuitry required for this task would be much simpler than what would be required for a neural pacemaker.

Although insulin oscillations persist in the perfused pancreas, the *in vivo* environment has additional factors that can be synchronizing aids. Small glucose oscillations, likely due to pancreas-liver interactions, have been measured with a period similar to that of the insulin oscillations [17,60–62]. These oscillations could help to synchronize the islets, as has been demonstrated *in vitro* [15,43,63]. There are also other factors released by postganglia neurons, such as ATP, that could assist in interislet synchronization [41,44]. Given the robustness of insulin oscillations across species, it is likely that interislet synchronization is mediated by several factors, providing robustness to the system.

## Conclusions

We have demonstrated that oscillations in islet activity, as measured through the intracellular Ca^2+^ concentration, can be entrained by the periodic application of the cholinergic agonist CCh. This is true for a range of stimulus periods from 2 to 10 min. In the case of the 2-min stimulus period there is often 2:1 entrainment, with one Ca^2+^ pulse for every other CCh stimulus. In the case of the 10-min stimulus period there is often 1:2 entrainment with two Ca^2+^ pulses per stimulus.

We have also demonstrated that a periodic stimulus is not needed to synchronize islets. Indeed, it was shown both experimentally and in computer simulations that randomly-timed CCh pulses are sufficient to synchronize islet activity, provided that the time between the CCh pulses is not too large. This suggests that, in vivo, a neural pacemaker is not necessary to synchronize the many islets within the pancreas; all that is needed is a neural system operating through pancreatic ganglia, that provides coordinated input to the islets. This would result in coordinated insulin secretion from the islet population and would lead to the rhythmic insulin levels observed in non-diabetic animals.

## Supporting information

S1 Fig Experimental setup. A piezoelectric pressure regulator facilitates accurate flow control of reagents from reservoir, through an inline flow sensor, to the microfluidic device. Conventional Ca^2+^ imaging with FURA-PE3 was employed with the relevant excitation filters and dichroic mirror as well as a CCD camera for fluorescence detection.
(PDF)

S2 Fig Data analysis work flow for production of spectrograms. (A) A sinusoidal wave is used to mimic a measured Ca^2+^ trace from a single islet. In this example, there is a constant background signal that gives rise to the elevated signal above baseline. The period and amplitude of the wave change with time. The period is 5 min during the first 30 min, 3 min during the next 30 min, and 4 min during the final 30 min. In those time spans, amplitude changes from 30 to 50 and then back down to 20. (B) A linear fit to the data in (A) was used to subtract the background signal producing a wave that is centered on y = 0. Simultaneously, the trace was smoothed by a single point linear interpolation of the data. (C) An STFT was used to analyze the data from (B) in 256 point long windows. The frequency data from each STFT was converted to period (min) and plotted on the y-axis. The next STFT window was moved 5-points later and the analysis repeated. The intensity of the peaks in the STFT was plotted in pseudocolor and shown by the scalebar on the right. In this example, the 5, 3, and 4 min period oscillations of the original trace are shown in the spectrogram with distinct 30 min long bands.
(PDF)

S3 Fig Islet oscillations are unsynchronized (1^st^ replicate). (A) The 1^st^ negative control experiment with 4 islets perfused with 11 mM glucose is shown. Gray lines indicate the individual islets and the black line indicates the mean [Ca^2+^]_i_. Neither the mean [Ca^2+^]_i_ nor the spectrogram (B) of the mean trace show any clear evidence of synchronization for the 90 min measurement.
(PDF)

S4 Fig Islet oscillations are unsynchronized (2^nd^ replicate). (A) The 2^nd^ negative control experiment with 4 islets perfused with 11 mM glucose is shown. Gray lines indicate the individual islets and the black line indicates the mean [Ca^2+^]_i_. Neither the mean [Ca^2+^]_i_ nor the spectrogram (B) of the mean trace show any clear evidence of synchronization for the 90 min measurement.
(PDF)

S5 Fig Islet oscillations are unsynchronized (3^rd^ replicate). (A) The 3^rd^ negative control experiment with 4 islets perfused with 11 mM glucose is shown. Gray lines indicate the individual islets and the black line indicates the mean [Ca^2+^]_i_. Neither the mean [Ca^2+^]_i_ nor the spectrogram (B) of the mean trace show any clear evidence of synchronization for the 90 min measurement.
(PDF)

S6 Fig Effect of CCh pulses with rest time of R = 2 on [Ca^2+^]_i_ oscillations (2^nd^ replicate). (A) The [Ca^2+^]_i_ traces (gray lines) of four islets and the average (black line) are shown when CCh pulses with rest durations of R = 2 min were applied. The timing of the CCh pulses is shown by the “x” at the top of the figure. The inset shows the 4 islets had natural periods ranging from 4–5 min prior to pulsing (open circles). During pulsing, oscillation periods transitioned to 2 min (filled circle) indicating 1:1 entrainment. The number of islets with identical oscillation periods before or during pulsing is indicated adjacent to the relevant circle. (B) The spectrogram of the average [Ca^2+^]_i_ from the 4 islets in (A) is shown. A prominent oscillation period band emerges at 2 min for the 38 min long CCh pulsing duration indicating synchronization among this islet group. The spectrogram did not show robust oscillation period bands outside the pulsing duration. Any >10 min oscillation period band is an artifact from the STFT data analysis.
(PDF)

S7 Fig Effect of CCh pulses with rest time of R = 2 on [Ca^2+^]_i_ oscillations (3^rd^ replicate). (A) The [Ca^2+^]_i_ traces (gray lines) of four islets and the average (black line) are shown when CCh pulses with rest durations of R = 2 min were applied. The timing of the CCh pulses is shown by the “x” at the top of the figure. The inset shows the 4 islets had natural periods ranging from ~3–10 min prior to pulsing (open circles). During pulsing, oscillation periods transitioned to ~4 min (filled circle) indicating 2:1 entrainment. The number of islets with identical oscillation periods before or during pulsing is indicated adjacent to the relevant circle. (B) The spectrogram of the average [Ca^2+^]_i_ from the 4 islets in (A) is shown. An oscillation period band emerges at ~4–5 min after 20 minutes of CCh pulsing indicating some islet synchronization. The >10 min oscillation period band is an artifact from the STFT data analysis.
(PDF)

S8 Fig Effect of CCh pulses with rest time of R = 5 on [Ca^2+^]_i_ oscillations (2^nd^ replicate). (A) The individual [Ca^2+^]_i_ from all 4 islets are shown in grey and the average is shown in black. The timing of the CCh pulses is shown by the “x”. The inset shows the natural period of these 4 islets ranged from 6.8–10.2 min (open circle), but all converged to 5 min during pulsing (filled circle) indicating 1:1 entrainment. (B) The synchronized response of the group is evident by the emergence of an albeit weaker oscillation period band at 5 min within the 55 min time span of pulsing in the spectrogram.
(PDF)

S9 Fig Effect of CCh pulses with rest time of R = 5 on [Ca^2+^]_i_ oscillations (3^rd^ replicate). (A) The individual [Ca^2+^]_i_ from all 4 islets are shown in grey and the average is shown in black. The timing of the CCh pulses is shown by the “x”. The inset shows the natural period of these 4 islets ranged from 4.2–7.0 min (open circle), but all converged to 5 min during pulsing (filled circle) indicating 1:1 entrainment. (B) The synchronized response of the group is evident by the emergence of an oscillation period band at 5 min within the 55 min time span of pulsing in the spectrogram.
(PDF)

S10 Fig Effect of CCh pulses with rest time of R = 10 on [Ca^2+^]_i_ oscillations (1^st^ replicate). (A) The [Ca^2+^]_i_ traces of the four islets present (grey lines) and their average (black line) over 90 min are shown with an “x” noting the timing of each CCh pulse. The inset shows that the islets had natural oscillation periods between 4.1 and 6.8 min (open circles) and transitioned to 5.0 min (1:2). The number of islets with identical oscillation periods before or during pulsing is indicated adjacent to the relevant circle. (B) The 5 min period band in the spectrogram of the mean Ca^2+^ trace depicts the synchronicity among these islets during the 50 min pulsing duration. After the final pulse at 70 min, oscillations diminished in magnitude.
(PDF)

S11 Fig Effect of CCh pulses with rest time of R = 10 on [Ca^2+^]_i_ oscillations (2^nd^ replicate). (A) The [Ca^2+^]_i_ traces of the four islets present (grey lines) and their average (black line) over 90 min are shown with an “x” noting the timing of each CCh pulse. The inset shows that 3/4 islets had natural oscillation periods between 5.1 and 6.8 min (open circles) and transitioned to 5.0 (1:2). The remaining islet had a >15 min natural oscillation period and transitioned into 1:1 entrainment. The number of islets with identical oscillation periods before or during pulsing is indicated adjacent to the relevant circle. (B) The prominent band at a period of 5 min in the spectrogram of the mean Ca^2+^ trace shows the high degree of synchronization among these islets during the 50 min pulsing duration. After the final pulse at 70 min, oscillations diminished in magnitude.
(PDF)

S12 Fig Effect of CCh pulses with rest time of R = 10 on [Ca^2+^]_i_ oscillations (3^rd^ replicate). (A) The [Ca^2+^]_i_ traces of the four islets present (grey lines) and their average (black line) over 80 min are shown with an “x” noting the timing of each CCh pulse. The inset shows that the islets had natural oscillation periods between 3.4 and 6.8 min (open circles) and transitioned to 5.0 (1:2) or 2.9 min oscillations (1:3) during pulsing (filled circles). The number of islets with identical oscillation periods before or during pulsing is indicated adjacent to the relevant circle. (B) As can be seen from the green band in the spectrogram of the average Ca^2+^ trace during the 50 min pulsing time span, these islets synchronized, albeit to a lesser degree than other islet groups in this experiment. After the final pulse at 70 min, oscillations diminished in magnitude.
(PDF)

S13 Fig Effect of CCh pulses with rest time of R = 10 on [Ca^2+^]_i_ oscillations (4^th^ replicate). (A) The [Ca^2+^]_i_ traces of the four islets present (grey lines) and their four islet average (black line) over 90 min are shown with an “x” noting the timing of each CCh pulse. The inset shows that the islets had natural oscillation periods of 6.8 or 10.2 min (open circles) and transitioned to 10.1 min oscillations (1:1) during pulsing (filled circles). The number of islets with identical oscillation periods before or during pulsing is indicated adjacent to the relevant circle. (B) The green 10 min oscillation period band in the spectrogram shows the synchronization of the doublet-like oscillations in among this islet group. The weaker ~5 min bands correspond to the intra doublet gaps in the average Ca^2+^ trace.
(PDF)

S14 Fig Randomly spaced CCh pulses synchronize islets (2^nd^ replicate). (A) The Ca^2+^ traces (grey lines) from a unique, representative experiment with a set of 4 islets are overlaid with the average [Ca^2+^]_i_ (black line). Each of the CCh pulses is shown by “x”. Although randomly spaced, the repeated CCh pulses produced a synchronized population of 4 islets as can be seen from the emergence of a green band in the spectrogram (B) of the 4 islet mean trace. The degree of synchronization rapidly diminished after the final pulse at 77 min.
(PDF)

S15 Fig Randomly spaced CCh pulses synchronize islets (3^rd^ replicate). (A) The Ca^2+^ traces (grey lines) from a unique, representative experiment with a set of 4 islets are overlaid with the average [Ca^2+^]_i_ (black line). Each of the CCh pulses is shown by “x”. Although randomly spaced, the repeated CCh pulses produced a synchronized population of 4 islets as can be seen from the emergence of a green band in the spectrogram (B) of the 4 islet mean trace.
(PDF)
