# Supplementary figures and images for: Synchronization of pancreatic islets by periodic or non-periodic muscarinic agonist pulse trains
Source: PLoS One. 2019 Feb 6;14(2):e0211832. doi: 10.1371/journal.pone.0211832 (PMC6364940; doi:10.1371/journal.pone.0211832)

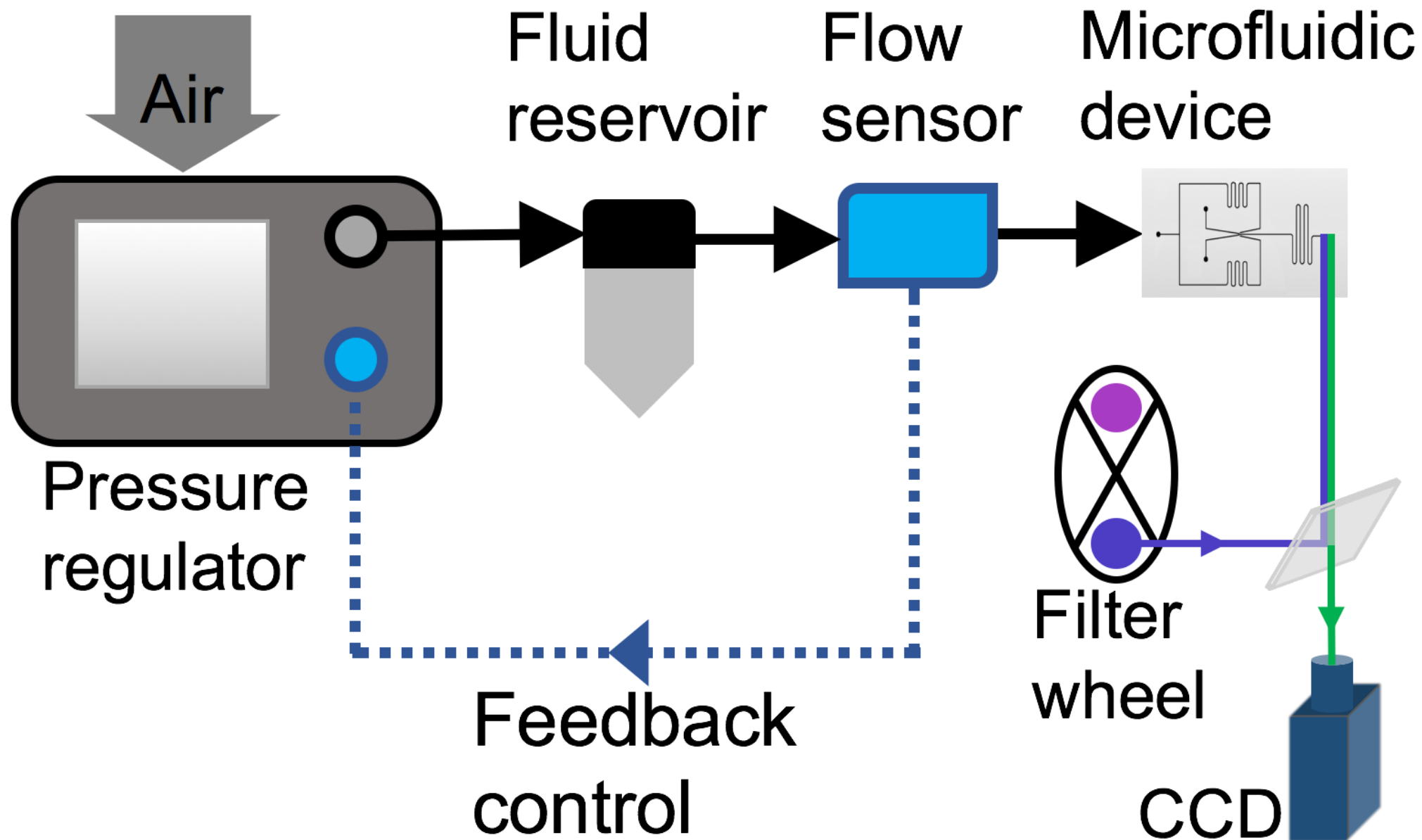

Supplement: S1 Fig — A piezoelectric pressure regulator facilitates accurate flow control of reagents from reservoir, through an inline flow sensor, to the microfluidic device. Conventional Ca2+ imaging with FURA-PE3 was employed with the relevant excitation filters and dichroic mirror as well as a CCD camera for fluorescence detection. (PDF) [file pone.0211832.s001.pdf]

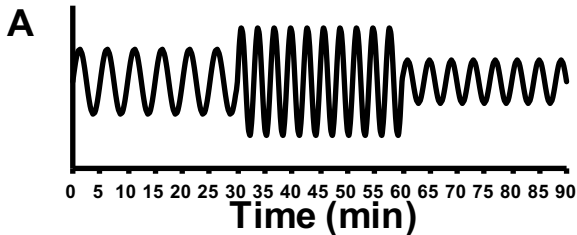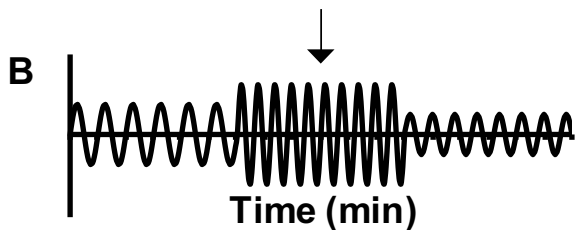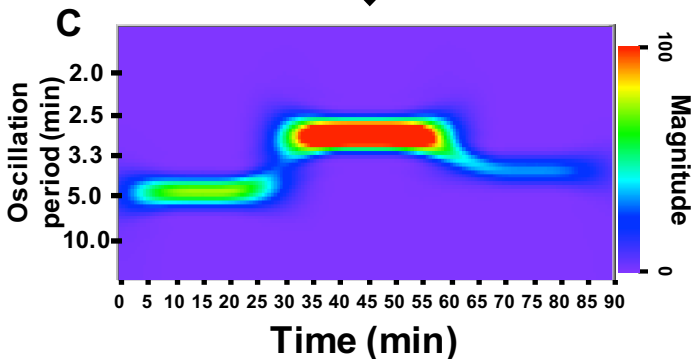

Supplement: S2 Fig — (A) A sinusoidal wave is used to mimic a measured Ca2+ trace from a single islet. In this example, there is a constant background signal that gives rise to the elevated signal above baseline. The period and amplitude of the wave change with time. The period is 5 min during the first 30 min, 3 min during the next 30 min, and 4 min during the final 30 min. In those time spans, amplitude changes from 30 to 50 and then back down to 20. (B) A linear fit to the data in (A) was used to subtract the background signal producing a wave that is centered on y = 0. Simultaneously, the trace was smoothed by a single point linear interpolation of the data. (C) An STFT was used to analyze the data from (B) in 256 point long windows. The frequency data from each STFT was converted to period (min) and plotted on the y-axis. The next STFT window was moved 5-points later and the analysis repeated. The intensity of the peaks in the STFT was plotted in pseudocolor and shown by the scalebar on the right. In this example, the 5, 3, and 4 min period oscillations of the original trace are shown in the spectrogram with distinct 30 min long bands. (PDF) [file pone.0211832.s002.pdf]

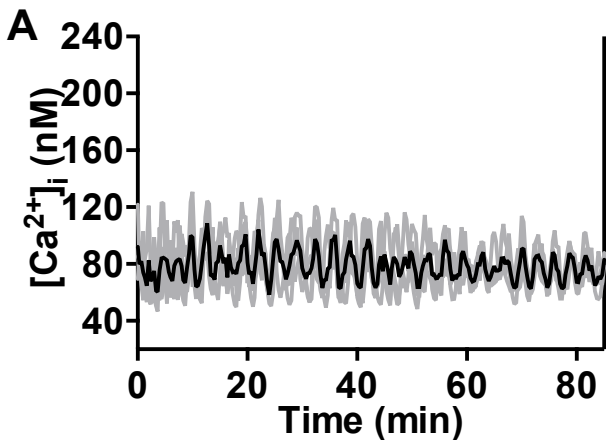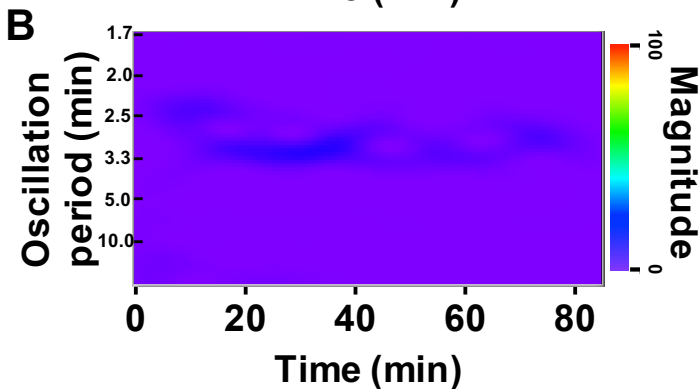

Supplement: S3 Fig — (A) The 1st negative control experiment with 4 islets perfused with 11 mM glucose is shown. Gray lines indicate the individual islets and the black line indicates the mean [Ca2+]i. Neither the mean [Ca2+]i nor the spectrogram (B) of the mean trace show any clear evidence of synchronization for the 90 min measurement. (PDF) [file pone.0211832.s003.pdf]

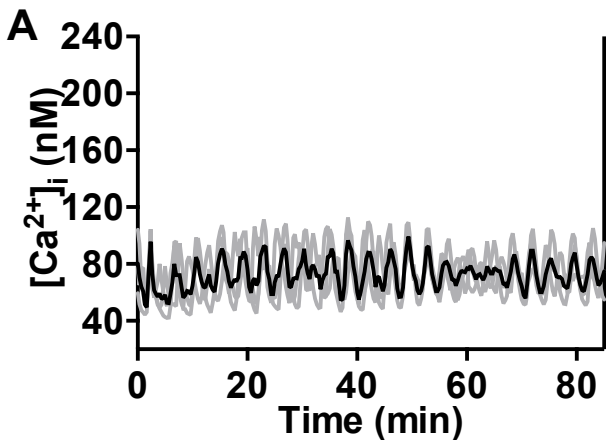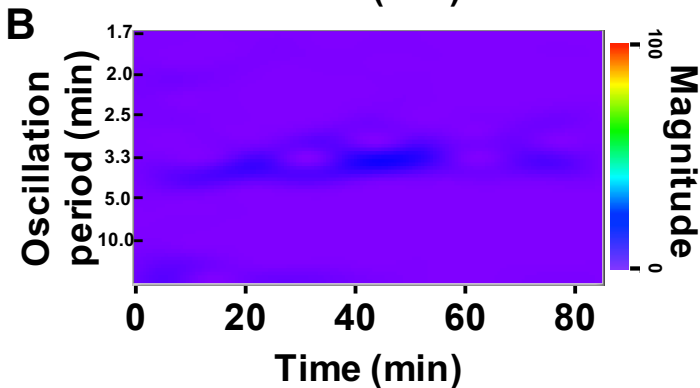

Supplement: S4 Fig — (A) The 2nd negative control experiment with 4 islets perfused with 11 mM glucose is shown. Gray lines indicate the individual islets and the black line indicates the mean [Ca2+]i. Neither the mean [Ca2+]i nor the spectrogram (B) of the mean trace show any clear evidence of synchronization for the 90 min measurement. (PDF) [file pone.0211832.s004.pdf]

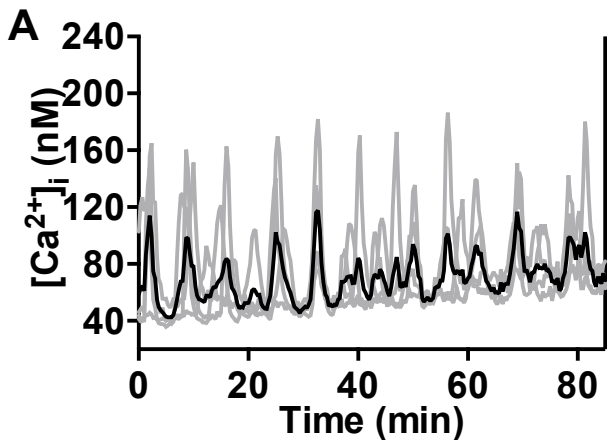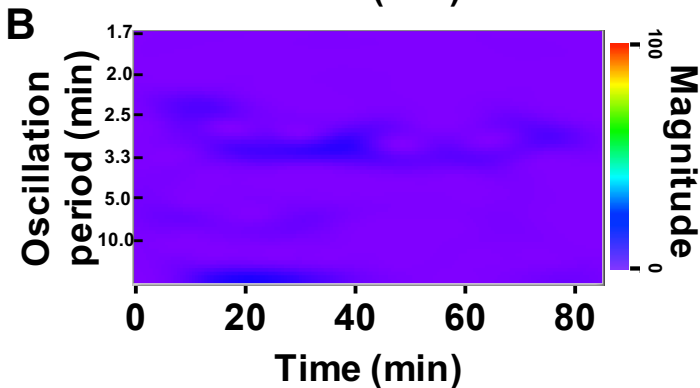

Supplement: S5 Fig — (A) The 3rd negative control experiment with 4 islets perfused with 11 mM glucose is shown. Gray lines indicate the individual islets and the black line indicates the mean [Ca2+]i. Neither the mean [Ca2+]i nor the spectrogram (B) of the mean trace show any clear evidence of synchronization for the 90 min measurement. (PDF) [file pone.0211832.s005.pdf]

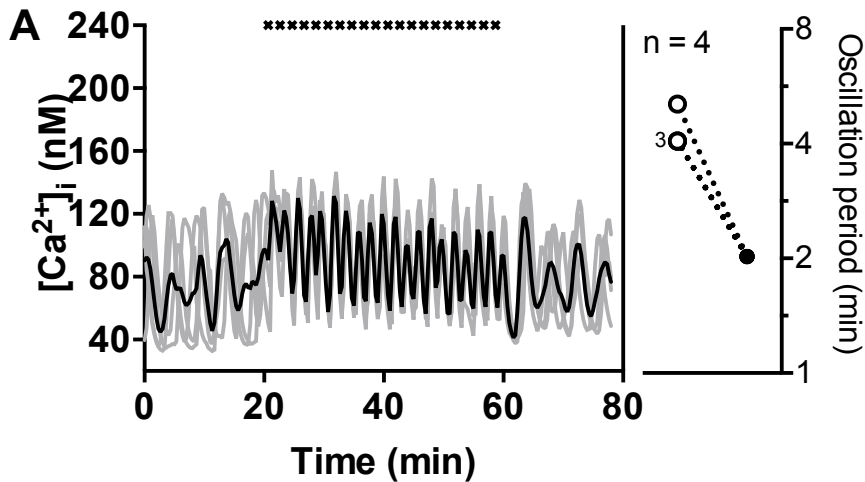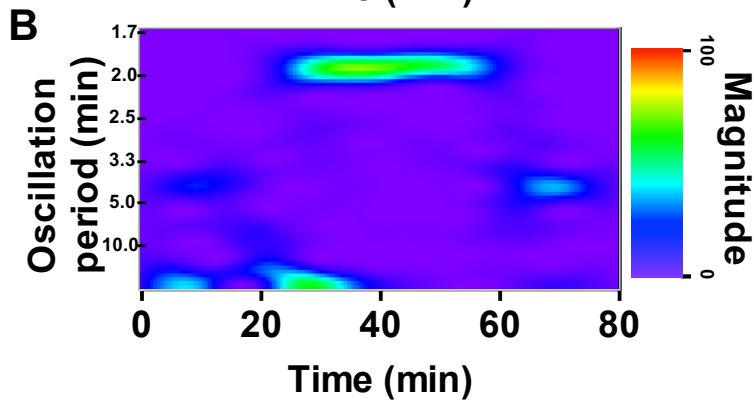

Supplement: S6 Fig — (A) The [Ca2+]i traces (gray lines) of four islets and the average (black line) are shown when CCh pulses with rest durations of R = 2 min were applied. The timing of the CCh pulses is shown by the “x” at the top of the figure. The inset shows the 4 islets had natural periods ranging from 4–5 min prior to pulsing (open circles). During pulsing, oscillation periods transitioned to 2 min (filled circle) indicating 1:1 entrainment. The number of islets with identical oscillation periods before or during pulsing is indicated adjacent to the relevant circle. (B) The spectrogram of the average [Ca2+]i from the 4 islets in (A) is shown. A prominent oscillation period band emerges at 2 min for the 38 min long CCh pulsing duration indicating synchronization among this islet group. The spectrogram did not show robust oscillation period bands outside the pulsing duration. Any >10 min oscillation period band is an artifact from the STFT data analysis. (PDF) [file pone.0211832.s006.pdf]

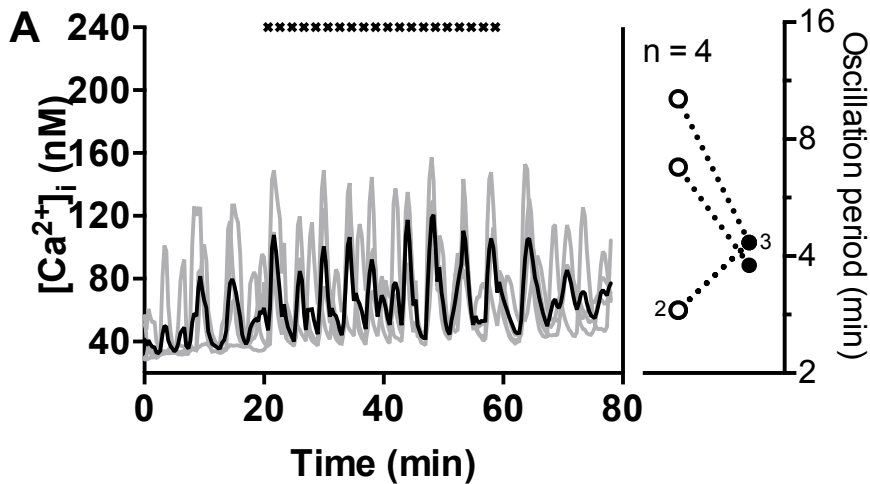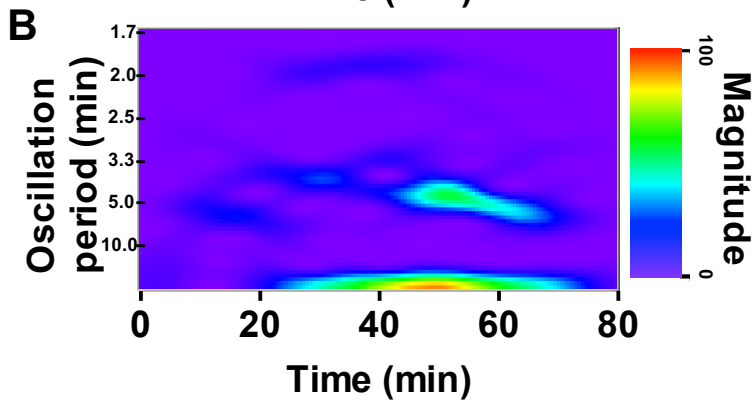

Supplement: S7 Fig — (A) The [Ca2+]i traces (gray lines) of four islets and the average (black line) are shown when CCh pulses with rest durations of R = 2 min were applied. The timing of the CCh pulses is shown by the “x” at the top of the figure. The inset shows the 4 islets had natural periods ranging from ~3–10 min prior to pulsing (open circles). During pulsing, oscillation periods transitioned to ~4 min (filled circle) indicating 2:1 entrainment. The number of islets with identical oscillation periods before or during pulsing is indicated adjacent to the relevant circle. (B) The spectrogram of the average [Ca2+]i from the 4 islets in (A) is shown. An oscillation period band emerges at ~4–5 min after 20 minutes of CCh pulsing indicating some islet synchronization. The >10 min oscillation period band is an artifact from the STFT data analysis. (PDF) [file pone.0211832.s007.pdf]

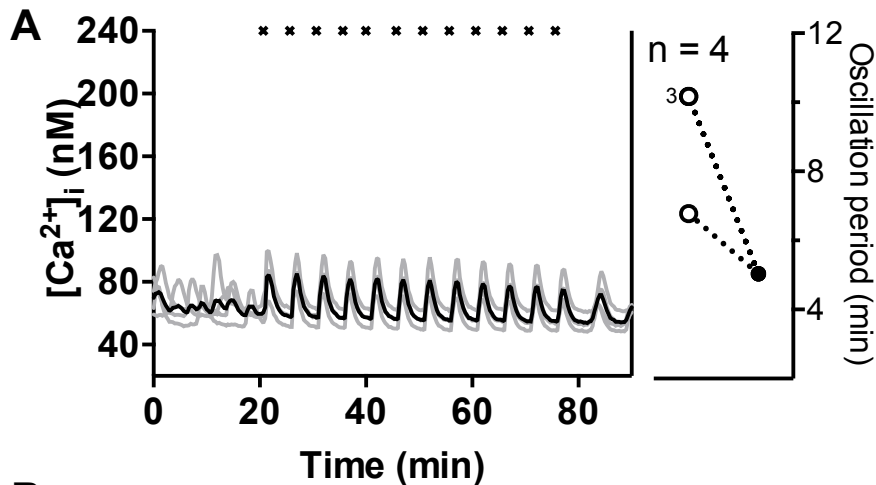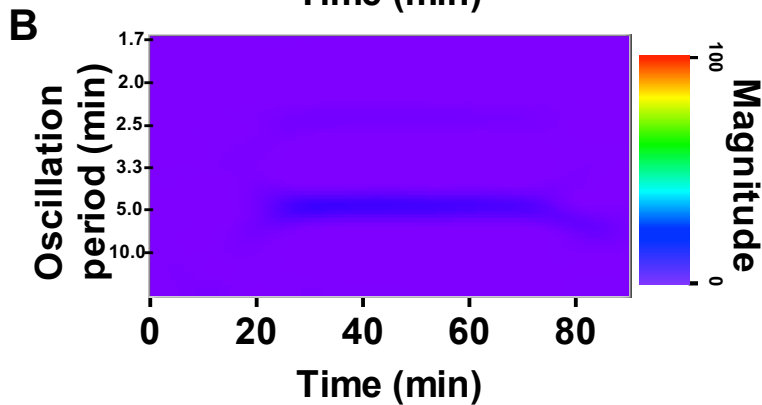

Supplement: S8 Fig — (A) The individual [Ca2+]i from all 4 islets are shown in grey and the average is shown in black. The timing of the CCh pulses is shown by the “x”. The inset shows the natural period of these 4 islets ranged from 6.8–10.2 min (open circle), but all converged to 5 min during pulsing (filled circle) indicating 1:1 entrainment. (B) The synchronized response of the group is evident by the emergence of an albeit weaker oscillation period band at 5 min within the 55 min time span of pulsing in the spectrogram. (PDF) [file pone.0211832.s008.pdf]

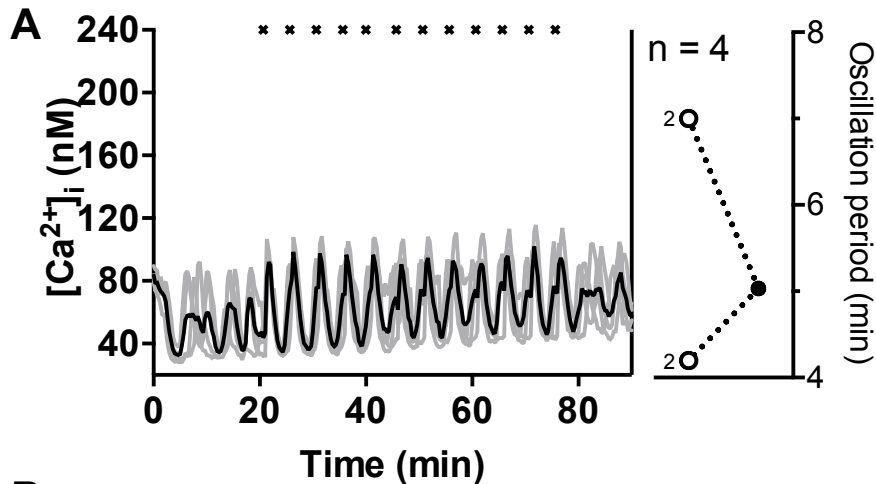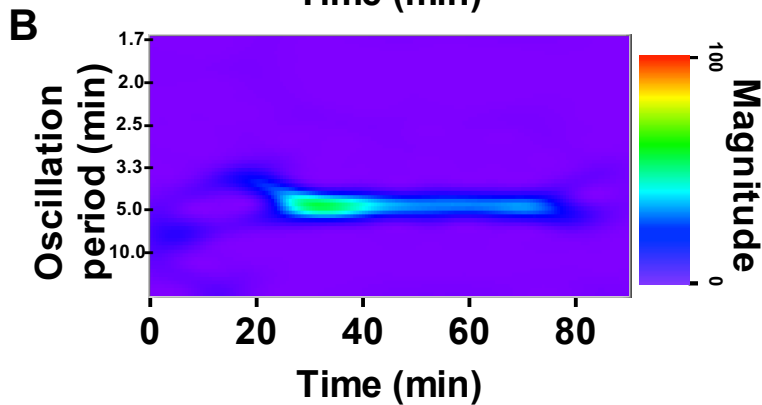

Supplement: S9 Fig — (A) The individual [Ca2+]i from all 4 islets are shown in grey and the average is shown in black. The timing of the CCh pulses is shown by the “x”. The inset shows the natural period of these 4 islets ranged from 4.2–7.0 min (open circle), but all converged to 5 min during pulsing (filled circle) indicating 1:1 entrainment. (B) The synchronized response of the group is evident by the emergence of an oscillation period band at 5 min within the 55 min time span of pulsing in the spectrogram. (PDF) [file pone.0211832.s009.pdf]

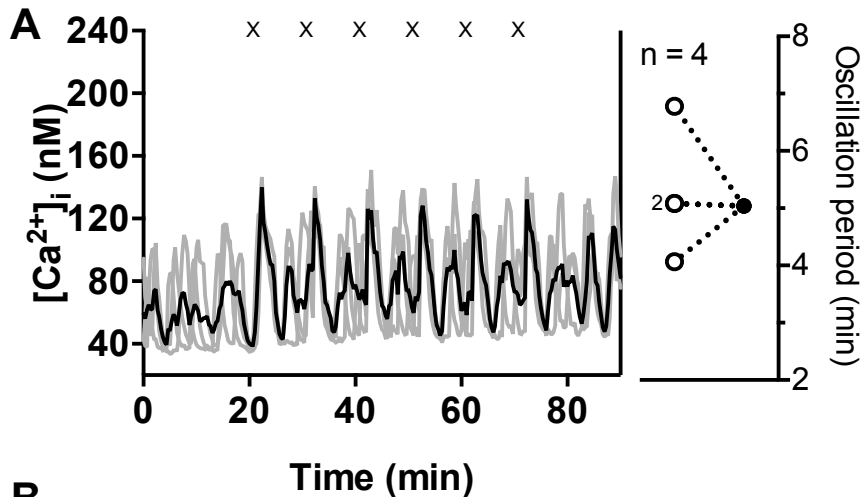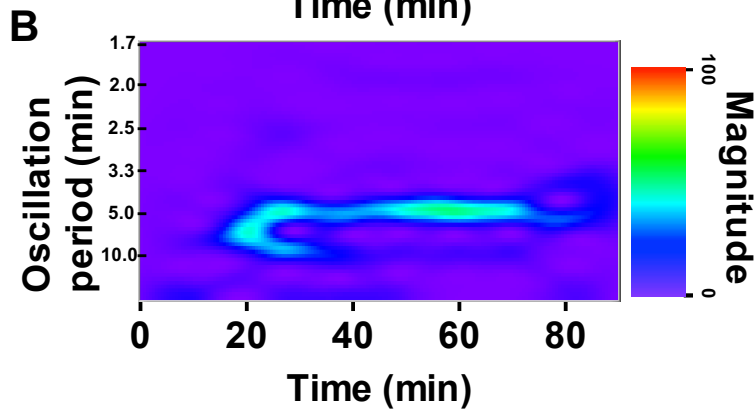

Supplement: S10 Fig — (A) The [Ca2+]i traces of the four islets present (grey lines) and their average (black line) over 90 min are shown with an “x” noting the timing of each CCh pulse. The inset shows that the islets had natural oscillation periods between 4.1 and 6.8 min (open circles) and transitioned to 5.0 min (1:2). The number of islets with identical oscillation periods before or during pulsing is indicated adjacent to the relevant circle. (B) The 5 min period band in the spectrogram of the mean Ca2+ trace depicts the synchronicity among these islets during the 50 min pulsing duration. After the final pulse at 70 min, oscillations diminished in magnitude. (PDF) [file pone.0211832.s010.pdf]

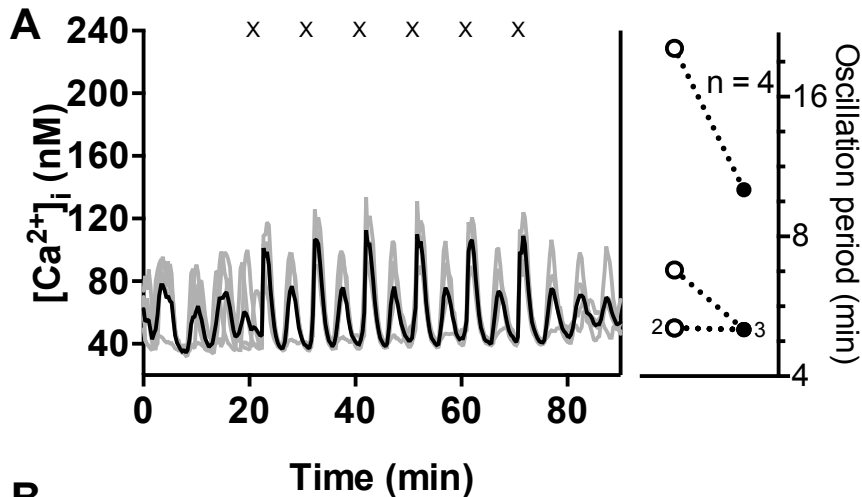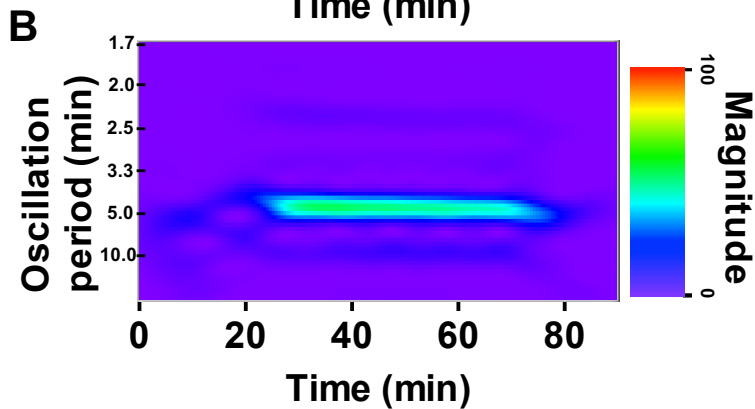

Supplement: S11 Fig — (A) The [Ca2+]i traces of the four islets present (grey lines) and their average (black line) over 90 min are shown with an “x” noting the timing of each CCh pulse. The inset shows that 3/4 islets had natural oscillation periods between 5.1 and 6.8 min (open circles) and transitioned to 5.0 (1:2). The remaining islet had a >15 min natural oscillation period and transitioned into 1:1 entrainment. The number of islets with identical oscillation periods before or during pulsing is indicated adjacent to the relevant circle. (B) The prominent band at a period of 5 min in the spectrogram of the mean Ca2+ trace shows the high degree of synchronization among these islets during the 50 min pulsing duration. After the final pulse at 70 min, oscillations diminished in magnitude. (PDF) [file pone.0211832.s011.pdf]

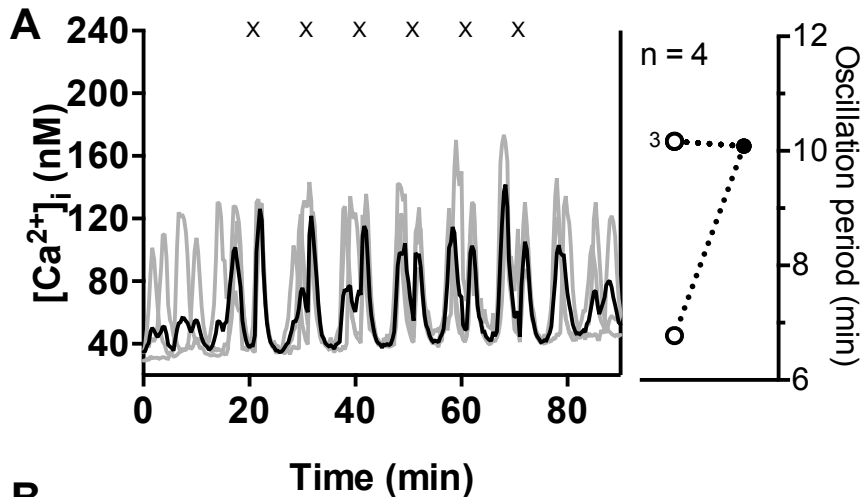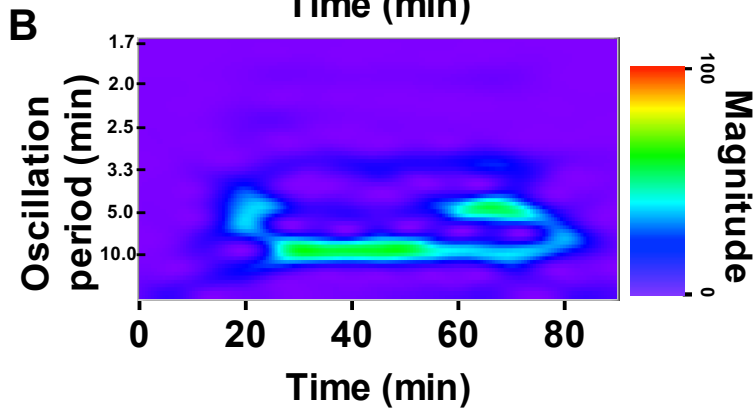

Supplement: S13 Fig — (A) The [Ca2+]i traces of the four islets present (grey lines) and their four islet average (black line) over 90 min are shown with an “x” noting the timing of each CCh pulse. The inset shows that the islets had natural oscillation periods of 6.8 or 10.2 min (open circles) and transitioned to 10.1 min oscillations (1:1) during pulsing (filled circles). The number of islets with identical oscillation periods before or during pulsing is indicated adjacent to the relevant circle. (B) The green 10 min oscillation period band in the spectrogram shows the synchronization of the doublet-like oscillations in among this islet group. The weaker ~5 min bands correspond to the intra doublet gaps in the average Ca2+ trace. (PDF) [file pone.0211832.s013.pdf]

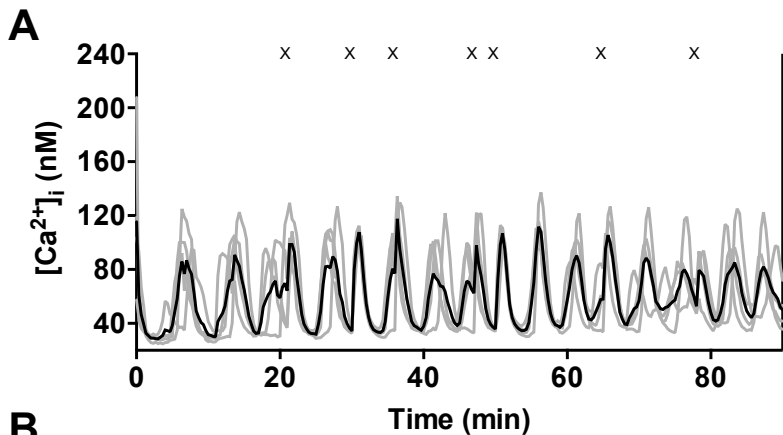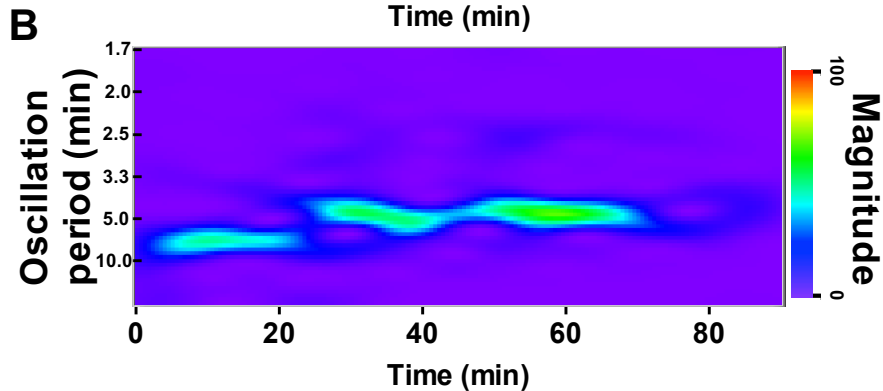

Supplement: S14 Fig — (A) The Ca2+ traces (grey lines) from a unique, representative experiment with a set of 4 islets are overlaid with the average [Ca2+]i (black line). Each of the CCh pulses is shown by “x”. Although randomly spaced, the repeated CCh pulses produced a synchronized population of 4 islets as can be seen from the emergence of a green band in the spectrogram (B) of the 4 islet mean trace. The degree of synchronization rapidly diminished after the final pulse at 77 min. (PDF) [file pone.0211832.s014.pdf]

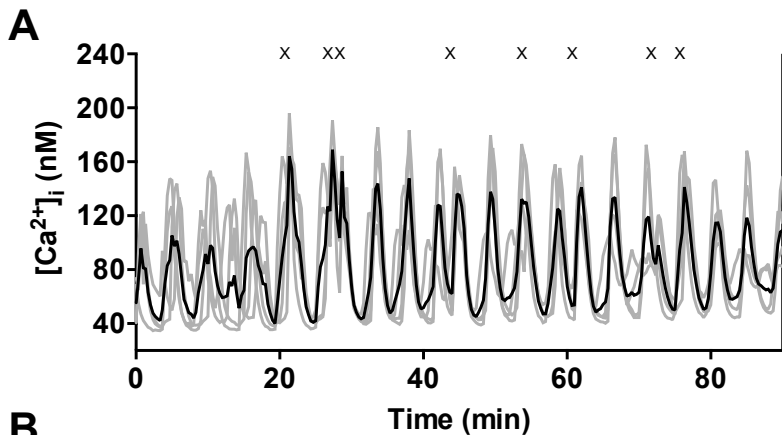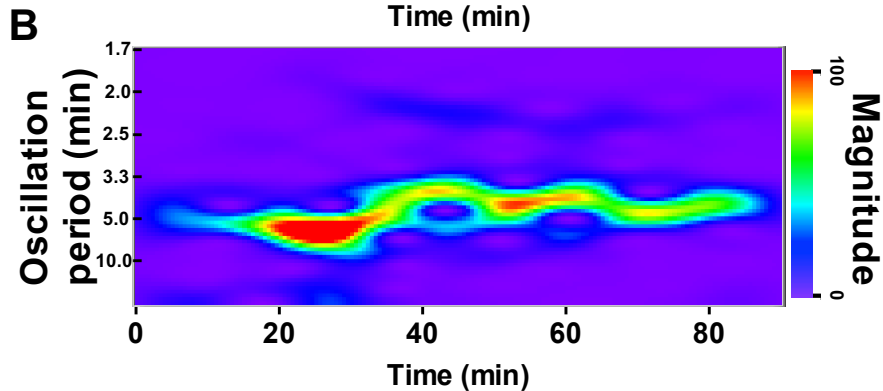

Supplement: S15 Fig — (A) The Ca2+ traces (grey lines) from a unique, representative experiment with a set of 4 islets are overlaid with the average [Ca2+]i (black line). Each of the CCh pulses is shown by “x”. Although randomly spaced, the repeated CCh pulses produced a synchronized population of 4 islets as can be seen from the emergence of a green band in the spectrogram (B) of the 4 islet mean trace. (PDF) [file pone.0211832.s015.pdf]
