# Supplementary material for: Synchronization of pancreatic islets by periodic or non-periodic muscarinic agonist pulse trains
Source: PLoS One. 2019 Feb 6;14(2):e0211832. doi: 10.1371/journal.pone.0211832 (PMC6364940; doi:10.1371/journal.pone.0211832)
Supplement: S12 Fig — (A) The [Ca2+]i traces of the four islets present (grey lines) and their average (black line) over 80 min are shown with an “x” noting the timing of each CCh pulse. The inset shows that the islets had natural oscillation periods between 3.4 and 6.8 min (open circles) and transitioned to 5.0 (1:2) or 2.9 min oscillations (1:3) during pulsing (filled circles). The number of islets with identical oscillation periods before or during pulsing is indicated adjacent to the relevant circle. (B) As can be seen from the green band in the spectrogram of the average Ca2+ trace during the 50 min pulsing time span, these islets synchronized, albeit to a lesser degree than other islet groups in this experiment. After the final pulse at 70 min, oscillations diminished in magnitude. (PDF) [file pone.0211832.s012.pdf]

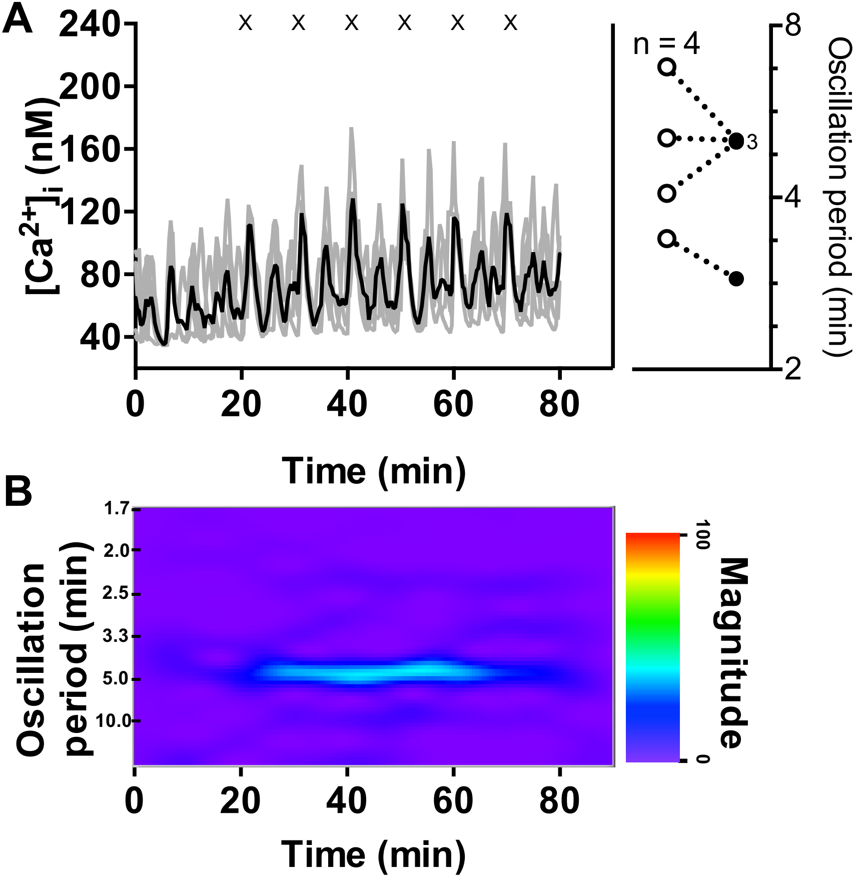

A
240
200
160
120
80
40
[Ca2+]i (nM)
x x x x x x
0
20
40
60
80
Time (min)
n = 4
3
8
4
2
Oscillation period (min)
B
1.7
2.0
2.5
3.3
5.0
10.0
Oscillation period (min)
0
20
40
60
80
Time (min)
100
0
Magnitude
